# 3′Nucleotidase/nuclease is required for *Leishmania infantum* clinical isolate susceptibility to miltefosine

**DOI:** 10.1016/j.ebiom.2022.104378

**Published:** 2022-11-30

**Authors:** Juliana B.T. Carnielli, Anuja Dave, Audrey Romano, Sarah Forrester, Pedro R. de Faria, Renata Monti-Rocha, Carlos H.N. Costa, Reynaldo Dietze, Ian A. Graham, Jeremy C. Mottram

**Affiliations:** a York Biomedical Research Institute, Department of Biology, University of York, United Kingdom; b Laboratório de Leishmanioses, Núcleo de Doenças Infecciosas, Universidade Federal do Espírito Santo, Vitória-ES, Brazil; c Centre for Novel Agricultural Products, Department of Biology, University of York, United Kingdom; d Laboratório de Pesquisas em Leishmanioses, Instituto de Doenças Tropicais Natan Portella, Universidade Federal do Piauí, Teresina-PI, Brazil; e Global Health & Tropical Medicine—Instituto de Higiene e Medicina Tropical—Universidade Nova de Lisboa, Lisbon, Portugal

**Keywords:** Miltefosine resistance, Visceral leishmaniasis, CRISPR-Cas9, 3'nucleotidase/nuclease, Prognostic marker

## Abstract

**Background:**

Miltefosine treatment failure in visceral leishmaniasis in Brazil has been associated with deletion of the miltefosine susceptibility locus (MSL) in *Leishmania infantum*. The MSL comprises four genes, 3′-nucleotidase/nucleases (*NUC1 and NUC2*); helicase-like protein (*HLP*); and 3,2-trans-enoyl-CoA isomerase (*TEI*).

**Methods:**

In this study CRISPR-Cas9 was used to either epitope tag or delete *NUC1*, *NUC2, HLP* and *TEI*, to investigate their role in miltefosine resistance mechanisms. Additionally, miltefosine transporter genes and miltefosine-mediated reactive oxygen species homeostasis were assessed in 26 *L. infantum* clinical isolates. A comparative lipidomic analysis was also performed to investigate the molecular basis of miltefosine resistance.

**Findings:**

Deletion of both *NUC1*, *NUC2* from the MSL was associated with a significant decrease in miltefosine susceptibility, which was restored after re-expression. Metabolomic analysis of parasites lacking the MSL or *NUC1* and *NUC2* identified an increase in the parasite lipid content, including ergosterol; these lipids may contribute to miltefosine resistance by binding the drug in the membrane. Parasites lacking the MSL are more resistant to lipid metabolism perturbation caused by miltefosine and *NUC1* and *NUC2* are involved in this pathway. Additionally, *L. infantum* parasites lacking the MSL isolated from patients who relapsed after miltefosine treatment were found to modulate nitric oxide accumulation in host macrophages.

**Interpretation:**

Altogether, these data indicate that multifactorial mechanisms are involved in natural resistance to miltefosine in *L. infantum* and that the absence of the 3'nucleotidase/nuclease genes *NUC1* and *NUC2* contributes to the phenotype.

**Funding:**

10.13039/501100000265MRC GCRF and 10.13039/501100006182FAPES.

**Research in context**

**Evidence before this study**

We have previously associated the presence of a genomic locus in *Leishmania infantum* parasite (MSL, Miltefosine Sensitivity Locus) with a positive response to miltefosine treatment, that could be used as a prognostic marker of therapeutic response to miltefosine in the treatment of visceral leishmaniasis (VL). The MSL contains four genes: *NUC1*, *NUC2*, *HLP* and *TEI*, however, the mechanism by which these genes sustain miltefosine susceptibility in *L. infantum* was not known.

**Added value of this study**

In this study we used CRISPR-Cas9 to genetically manipulate a clinical isolate of *L. infantum* with MSL^+^ locus in their genomes, thus generating mutants that allowed us to investigate, separately or in combination, the role of the 4 genes of the MSL locus. Using these mutant cell lines and clinical isolates from VL patients with different miltefosine treatment outcomes, we found that the genes encoding for the 3′-nucleotidase/nucleases (*NUC1* and *NUC2*) are the two genes involved in miltefosine susceptibility. We likewise found that the absence of *NUC1* and *NUC2* allow the parasite to modulate lipid metabolism perturbation caused by miltefosine.

**Implications of all the available evidence**

Our study showed that 3′-nucleotidase/nucleases are the key components of the prognostic marker MSL involved in miltefosine susceptibility. Our work brings new insights into miltefosine resistance in *Leishmania*, which can contribute to future drug development.

## Introduction

Globally distributed and closely related to poverty, the leishmaniases are neglected tropical diseases with more than 1 billion people at risk of infection.^1^ Visceral leishmaniasis (VL), with fatality rates up to 95% if left untreated, is the most severe form of a complex of leishmaniasis diseases and is caused by *Leishmania donovani* and *L. infantum* (synonymy with *Leishmania chagasi*). The number of new VL cases is estimated at 50,000–90,000 worldwide annually and most of these cases are reported in East Africa, India and Brazil.^1^ Leishmaniasis control relies primarily on chemotherapy, nevertheless, current therapies have severe shortcomings such as prolonged and painful applications of poorly tolerated toxic drugs. Furthermore, its treatment is hampered by the fact that there are few candidates in the clinical trials pipeline^2^, ^3^, ^4^, ^5^ and emergence of resistance to first-line drugs has a significant impact on therapy for this important parasitic disease.

In this scenario, the identification of an effective and safe oral drug miltefosine (hexadecylphosphocholine) was considered an important advance in leishmaniases control. Miltefosine, a phospholipid analog developed initially as an anticancer drug,^6^ is currently the only oral drug available for treatment of VL. When miltefosine was first introduced in India in 2002 to treat VL, caused by *L. donovani*, it exhibited a clinical cure of about 94%.^7^^,^^8^ Although miltefosine has played an essential role in the kala-azar elimination programme, a reduction in its efficacy has been observed after extensive use in the Indian subcontinent over almost two decades.^9^, ^10^, ^11^ The mechanism behind miltefosine resistance has been mainly studied in resistant cell lines generated *in vitro*, which has associated this phenotype with a decrease in intracellular drug accumulation: due to the defective inward drug translocation,^12^, ^13^, ^14^, ^15^, ^16^ and the over-expression of ABC transporters that are responsible for drug efflux.^17^^,^^18^ These traits were also found in some clinical isolates from Indian and Mediterranean regions^19^, ^20^, ^21^ as well as in *Leishmania braziliensis*.^22^

Studies have also shown that alterations in fatty acid and sterol biosynthesis impact the membrane lipid composition in miltefosine-resistant *L. donovani*, which could alter membrane fluidity and permeability and therefore affect the drug–membrane interaction.^23^^,^^24^ A chemical mutagenesis screening additionally associated mutations in genes involved in lipid metabolism (fatty acid elongase, long-chain fatty acyl CoA ligase and glycerol phosphoryl phosphodiesterase) with miltefosine resistance in *L. infantum*.^16^ Furthermore, overexpression of lipase precursor-like protein, theoretically involved in free fatty acid metabolism via the beta oxidation pathway, has also been associated with lower miltefosine susceptibility in *L. donovani* due to higher ability to control drug-induced oxidative stress and host macrophage modulation.^25^^,^^26^ The ability to manage drug-mediated cell death^27^, ^28^, ^29^ and drug-induced oxidative stress^30^ has been observed in miltefosine-resistant *Leishmania* parasites, and more recently, overexpression of the iron superoxide dismutase has been related with reactive oxygen species (ROS) scavenging mechanism, protecting parasite from ROS-induced damage.^31^

The least favourable miltefosine efficacy scenario was revealed by a phase II trial in Brazil, where cure rate was only 60% in VL caused by *L. infantum*.^32^ The *in vitro* miltefosine susceptibility profile of these isolates exhibited a positive correlation with the clinical outcome, indicating that therapeutic failure is associated with natural miltefosine resistance.^32^ Subsequently, it was found that deletion of the MSL (miltefosine susceptible locus) on chromosome 31 of the parasite was significantly correlated with miltefosine treatment failure.^33^ Nevertheless, a recent study was unable to demonstrate a correlation between the absence of MSL, in *L. infantum* isolates from a collection, and the *in vitro* resistance phenotype,^34^ identified in our Phase 2 clinical trial well characterized isolates.^32^^,^^33^

The MSL, highlighted as a promising molecular marker to guide an accurate clinical practice approach, was the focus of this study aimed to investigate the underlying molecular resistance mechanisms to miltefosine. Herein, we demonstrate that the 3'nucleotidase/nuclease is the MSL component that plays a role in susceptibility of *L. infantum* to miltefosine. Moreover, we demonstrate that resistant parasites are less susceptible to lipid metabolism perturbation caused by miltefosine and that the MSL and more specifically 3'nucleotidase/nuclease are involved in this pathway. Finally, we showed that *L. infantum* isolated from relapsed patients modulates nitric oxide accumulation in the host macrophage.

## Methods

### *Leishmania infantum* isolates

The *Leishmania* isolates were obtained by bone marrow aspirates from 26 patients enrolled in a clinical trial designed to evaluate the efficacy and toxicity of miltefosine in the treatment of VL in Brazil – 14 isolates from patients who showed cure after the six months follow up, and 12 isolates from patients who relapsed during the follow up.^32^ These clinical isolates were identified as *L. infantum* based on a PCR-RFLP assay.^35^

### Ethics

Ethical clearance for the use of the *L. infantum* clinical isolates was granted by the institutional review board at the Centro de Ciências da Saúde, Universidade Federal do Espírito Santo (CEP-066/2007), Brazil. All isolates and its use were also registered in SisGen (*Sistema Nacional de Gestão do Patrimonio Genético* - Brazil) under the identifier A790812.

### Cell culture

The promastigote stage of *L. infantum* was maintained in culture at 25 °C in complete SDM79 medium (Gibco, ThermoFisher Scientific): supplemented with 10% heat-inactivated fetal bovine serum (hi-FBS) (Gibco, ThermoFisher Scientific), 10 μM 6-biopterin (Sigma–Aldrich), and 100 U penicillin – 100 μg mL^−1^ streptomycin (Sigma–Aldrich), pH 7.2. The RAW264.7 macrophage cell line (RRID: CVCL_0493) was maintained in culture in RPMI 1640 medium supplemented with 10% hi-FBS and 10 mM l-glutamine (Gibco, ThermoFisher Scientific) at 37 °C, in an atmosphere of 5% CO_2_.

### Miltefosine transporter (MT) and β-subunit Ros3 (β-Ros3) gene expression

Total RNA was obtained from 10^8^ promastigotes in the mid-log phase of growth using the TRIzol™ reagent (Invitrogen™, ThermoFisher Scientific) following the manufacturer's instructions. The extracted RNA was then treated with Turbo DNA-free™ kit (Invitrogen™, ThermoFisher Scientific) to remove genomic DNA contamination. cDNA from promastigotes was synthesised using High Capacity cDNA Reverse Transcription Kit (Applied Biosystems™, ThermoFisher Scientific) and oligo(dT)18 primers as described by the manufacturer. The sequences of the primers used to quantify MT, β-Ros3 and the endogenous normaliser GAPDH (glyceraldehyde phosphate dehydrogenase) are detailed in Supplementary Table S1. Relative quantification was performed by Real-time PCR in triplicate in 20 μL volumes using the SYBR™ Green qPCR SuperMix (Invitrogen™, ThermoFisher Scientific) in an Applied Biosystem 7500 Fast system. Reactions were run using the following thermal profile: initial denaturation at 95 °C for 10 min followed by 40 cycles with denaturation at 95 °C for 15 s, annealing/extension at 60 °C for 1 min. The PCR was followed by a melt curve analysis to ascertain that the expected products were amplified. The relative amount of PCR products generated from each primer set was determined based on the threshold cycle (Ct) value and amplification efficiencies. The gene expression levels of MT and β-Ros3 were determined for *L. infantum* clinical isolates using the 2^−ΔΔCt^ method in comparison with the expression levels of reference Brazillian *L. infantum* strain MHOM/BR/74/PP75.

### Gene dose and SNPs (single nucleotide polymorphisms) and InDels (insertion-deletion polymorphism) variants of MT and β-Ros3

The whole genome sequencing generated previously^33^ was reanalysed to confirm the gene dose of MT (LINF_130020800) and β-Ros3 (LINJ_320010400), as well as for the presence of SNPs and InDels in these genes.

### Measurement of reactive oxygen species (ROS) accumulation in promastigote

Promastigotes of *L infantum* at 10^7^ cells mL^−1^ were incubated with varying miltefosine concentrations for different lengths of time. Intracellular ROS (H_2_O_2_, HO•, ROO• and ONOO^−^) and mitochondrial superoxide (•O_2_^-^) accumulation was measured using the cell permeant H_2_DCFDA and MitoSOX™ dyes (Invitrogen™, ThermoFisher Scientific), respectively. After drug exposure, 2 × 10^6^ parasites were washed twice at 2000 g for 5 min with Hank's Balanced Salt Solution (HBSS) at 4 °C and resuspended in 200 μL of 10 μM H_2_DCFDA or 3 μM MitoSOX™ in HBSS. Cells were incubated for 40 min at 25 °C and after washed twice with HBSS, under the same conditions previously described, resuspended in 500 μL of HBSS (for H_2_DCFDA dye preparation, 0.5 μg mL^−1^ of propidium iodide (PI) was added for live/dead cell discrimination). Cells were analysed for FACS using AttuneTM NxT Acoustic Focusing Cytometer (v.2.5.391.2) and the data were analysed using FlowJo™ 10.6.2 software.

### Measurement of ROS and nitric oxide (NO) accumulation in *L. infantum*-infected macrophage

Stationary phase *L. infantum* promastigotes were used to infect the RAW264.7 macrophage cell line. Prior to infection the parasites were stained with CellTrace™ (Invitrogen™, ThermoFisher Scientific) according to manufacturer instructions. Briefly, parasites were washed once at 2000 g for 5 min with PBS at room temperature and then, 2 × 10^7^ cells mL^−1^ were incubated for 20 min at 25 °C with 1 μM of CellTrace™ Far Red in PBS. The reaction was quenched by adding medium supplemented with 10% hi-FBS. Cells were pelleted at 2000 g for 5 min and resuspended in RPMI 1649 medium. Before proceeding with the infection, parasite staining was checked by flow cytometry (for all experiments more than 98% of cells were successfully stained).

RAW264.7 macrophages were plated on 96 wells plate (1.25 × 10^5^ cells in each well) and after 4 h incubation at 37 °C, in an atmosphere of 5% CO_2_, the macrophages were infected with the stained parasites (5 parasites per macrophage). After 24 h incubation at 37 °C, in an atmosphere of 5% CO_2_, the extracellular parasites were removed by gently washing with RPMI 1640 medium. Macrophages were then treated with 0 or 5 μM of miltefosine for additional 24 h incubation at the same conditions as above. Intracellular ROS (H_2_O_2_, HO•, ROO• and ONOO^−^), mitochondrial superoxide and NO accumulation were measured using the cell-permeant H_2_DCFDA, MitoSOX™, and DAF-FM dyes (Invitrogem™, ThermoFisher Scientific), respectively. After drug exposure, macrophages were detached from the plate by incubation at 37 °C for 10 min with PBS supplemented with 3 mM EDTA and 10 mM glucose, followed by successive pipetting. The cells were then washed twice at 2000 g for 5 min with HBSS at 4 °C and resuspended in 200 μL of 10 μM H_2_DCFDA or 3 μM MitoSOX™ or 5 μM DAF-FM in HBSS. Cells were incubated for 30 min at 37 °C (H_2_DCFDA and DAF-FM) or at 25 °C (MitoSOX™) and after washed twice with HBSS, resuspended in 500 μL of HBSS (for H_2_DCFDA and DAF-FM dyes preparation, 0.25 μg/mL of PI was added for live/dead cell discrimination). Cells were analysed for FACS using AttuneTM NxT Acoustic Focusing Cytometer (v.2.5.391.2) and the data were analysed using FlowJo™ 10.6.2 software.

### Generation of a *L. infantum* cell line expressing T7 RNA polymerase and Cas9 endonuclease

The clone 1 from the *L. infantum* isolate MHOM/BR/06/MA01A (MA01A-C1) that contains the MSL locus, was used to generate the *L. infantum* T7/Cas9 cell line. This isolate: (i) was obtained before the miltefosine treatment from a Brazilian VL patient who was cured after the treatment; (ii) and showed a susceptible *in vitro* phenotype to miltefosine (IC_50_ of 5.1 ± 0.3 μM and 2.7 ± 0.9 μM for amastigote and promastigote forms, respectively). For expression of T7 RNA polymerase and Cas9 endonuclease in *L. infantum*, we used a single-marker (HYG, hygromycin) expression plasmid (pTB010, Supplementary Fig. S1). This allowed the expression of hSpCas9 and T7RNA polymerase incorporating homologous arm for integration into the ribosomal locus of *Leishmania* spp. (pTB010, derived from pTB007,^36^ was kindly provided by Tom Beneke and Eva Gluenz from University of Oxford). Briefly, 10^7^ MA01A-C1 promastigotes were transfected with 10 μg of the linearised pTB010 plasmid (SwaI, New England Biolabs), using P3 Primary Cell 4D-Nucleofector (Lonza): one pulse with program FI-115 in a final volume of 110 μL. Electroporated cells were immediately transferred into pre-warmed medium and after 16 h recovering, they were selected and cloned in complete SMD79 solid medium supplemented with 150 μg mL^−1^ of hygromycin.

### *L. infantum* genome editing by CRISPR-Cas9

The MSL genes were deleted (Fig. 3a) and tagged at C-terminus, using the parental cell line expressing T7 RNA polymerase and Cas9 endonuclease, according to the genome editing toolkit for kinetoplastids developed by Beneke et al., 2017.^36^ The primers used to generate single-guide RNA (sgRNA) and repair templates were designed using LeishGEdit (http://leishgedit.net/). When LeishGEdit could not be used, the Eukaryotic Pathogen CRISPR guide RNA/DNA Design Tools (http://grna.ctegd.uga.edu) was used to identify guide RNA sequences, using the default parameters (SpCas9: 20 nt gRNA, NGG PAM on 3'end), and the primers were manually designed. sgRNA were designed to introduce a double strand break (DSB) downstream of the target gene, for both C-terminal endogenous tagging and null knockout mutant, for which a second guide was designed for DSB upstream the target. The repair template was designed to contain an extra 30 nucleotides for homology immediately adjacent to the DSB on DNA. To generate the null mutant cell lines two distinctive repair templates, differing only in the resistance marker, were used. 10^7^ parental T7/Cas9 MA01A-C1 promastigotes were transfected with the pooled sgRNA and repairs template, using P3 Primary Cell 4D-Nucleofector (Lonza): one pulse with program FI-115 in a final volume of 110 μL. Electroporated cells were immediately transferred into a pre-warmed medium. After 16 h recovery, they were selected and cloned in complete SMD79 solid medium supplemented with 150 μg mL^−1^ of blasticidin and/or 75 μg mL^−1^ of puromycin. Genomic DNA from recovered clones were then extracted and diagnostic PCRs were performed. Primers used to generate sgRNA and repair templates, as well as the ones used for diagnostic PCRs are available in Supplementary Table S1.

**Fig. 1:**
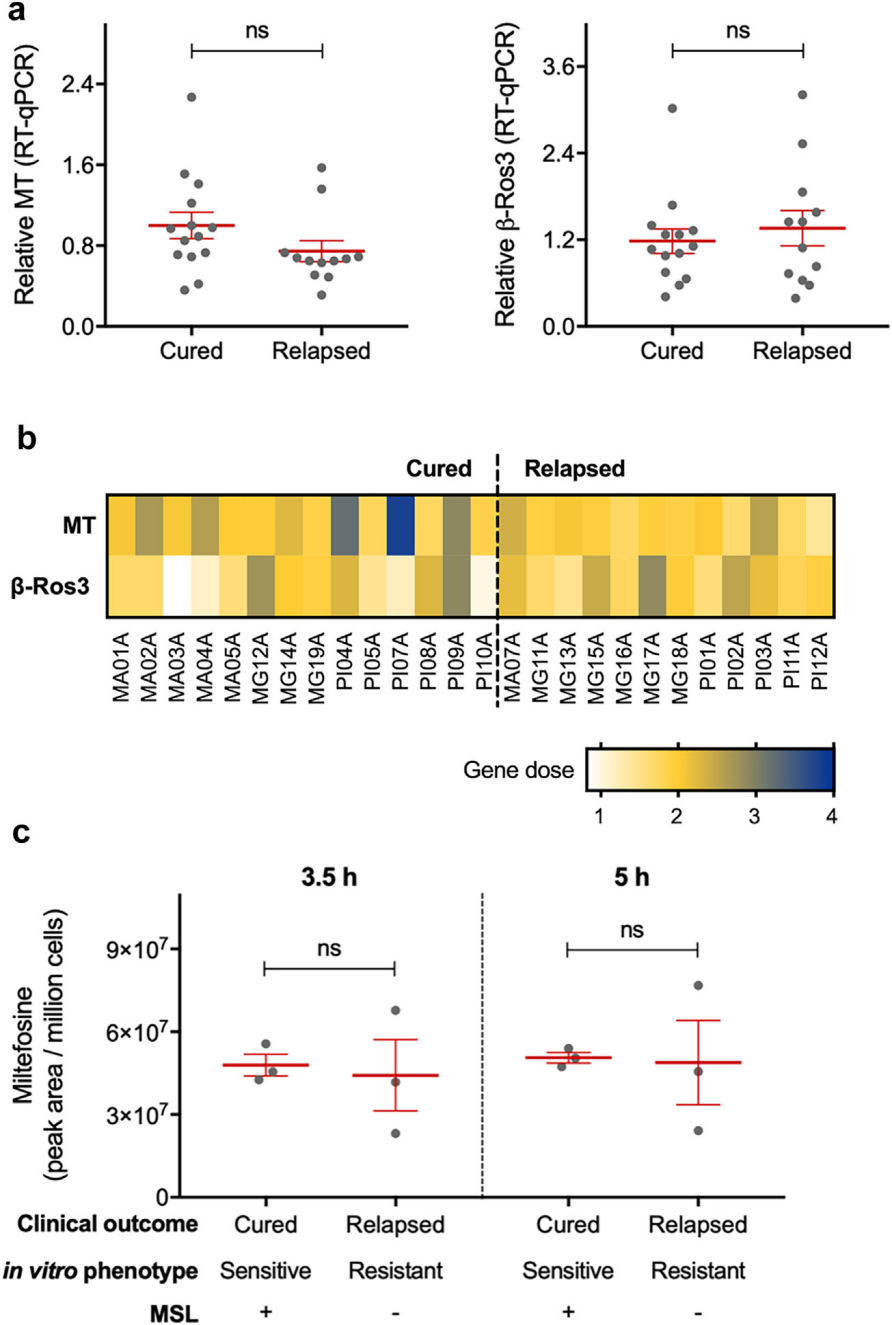
**Miltefosine transporter and its β-subunit Ros3 profile in *L. infantum* field isolates**. (a) Relative gene expression quantification for miltefosine transporter (MT, LINF_130020800) and its β-subunit Ros3 (β-Ros3, LINJ_320010400) in 26 *L. infantum* isolates (14 from group cured and 12 from group relapsed). (b) Gene dose of miltefosine transporter and its β-subunit Ros3 calculated from whole genome sequencing data. (c) Intracellular accumulation of miltefosine, measured by lipidomic analysis, in six *L. infantum* isolates after 3.5 h or 5.0 h of incubation with 10 μM miltefosine. Each grey dot represents the mean of individual *L. infantum* isolate from three independent experiments performed in triplicate. Mean and ±SEM for cured and relapsed groups are shown in red. P values were calculated using unpaired two-tailed Student's t-tests comparing the group cured with relapsed (ns, not significant).

**Fig. 2:**
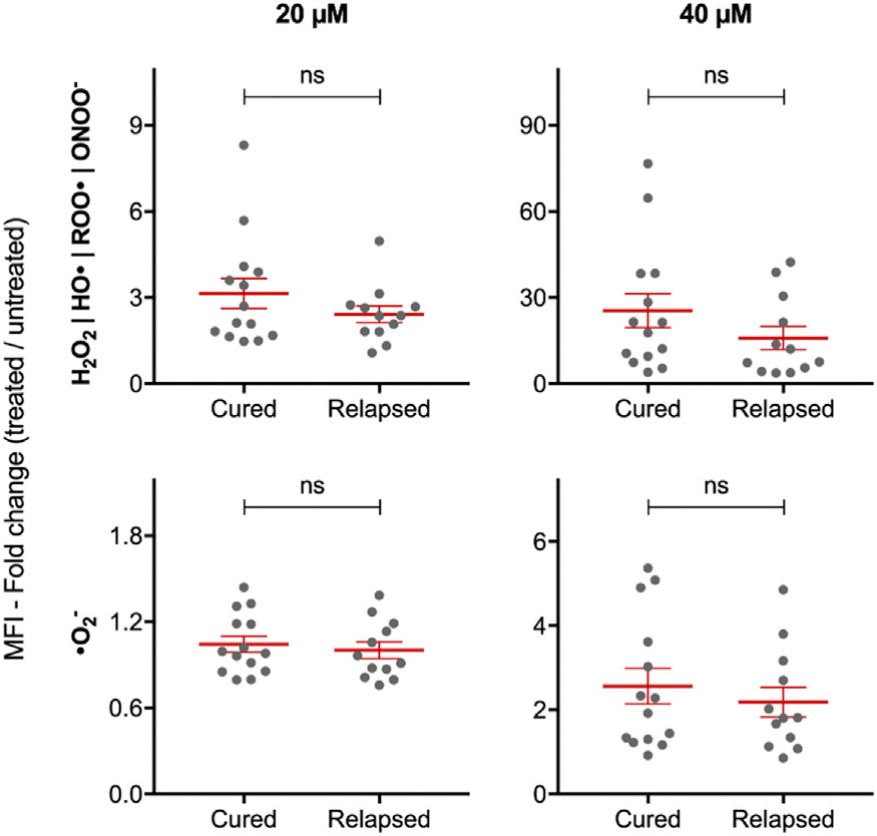
**Control of redox homeostasis by *L. infantum* promastigotes under miltefosine pressure**. 26 *L. infantum* isolates (14 from group cured and 12 from group relapsed) were treated with 0, 20 or 40 μM of miltefosine. The reactive oxygen species were measured using H_2_DCFDA (H_2_O_2_, HO•, ROO• and ONOO^−^) and MitoSOX™ (•O_2_^-^) after 4 and 24 h in the presence of miltefosine, respectively. Each grey dot represents the mean of individual *L. infantum* isolates from three independent experiments performed in triplicate. Mean ± SEM for cured and relapsed groups are shown in red. P values were calculated using unpaired two-tailed Student's t-tests comparing the group cured with relapsed (ns, not significant).

**Fig. 3:**
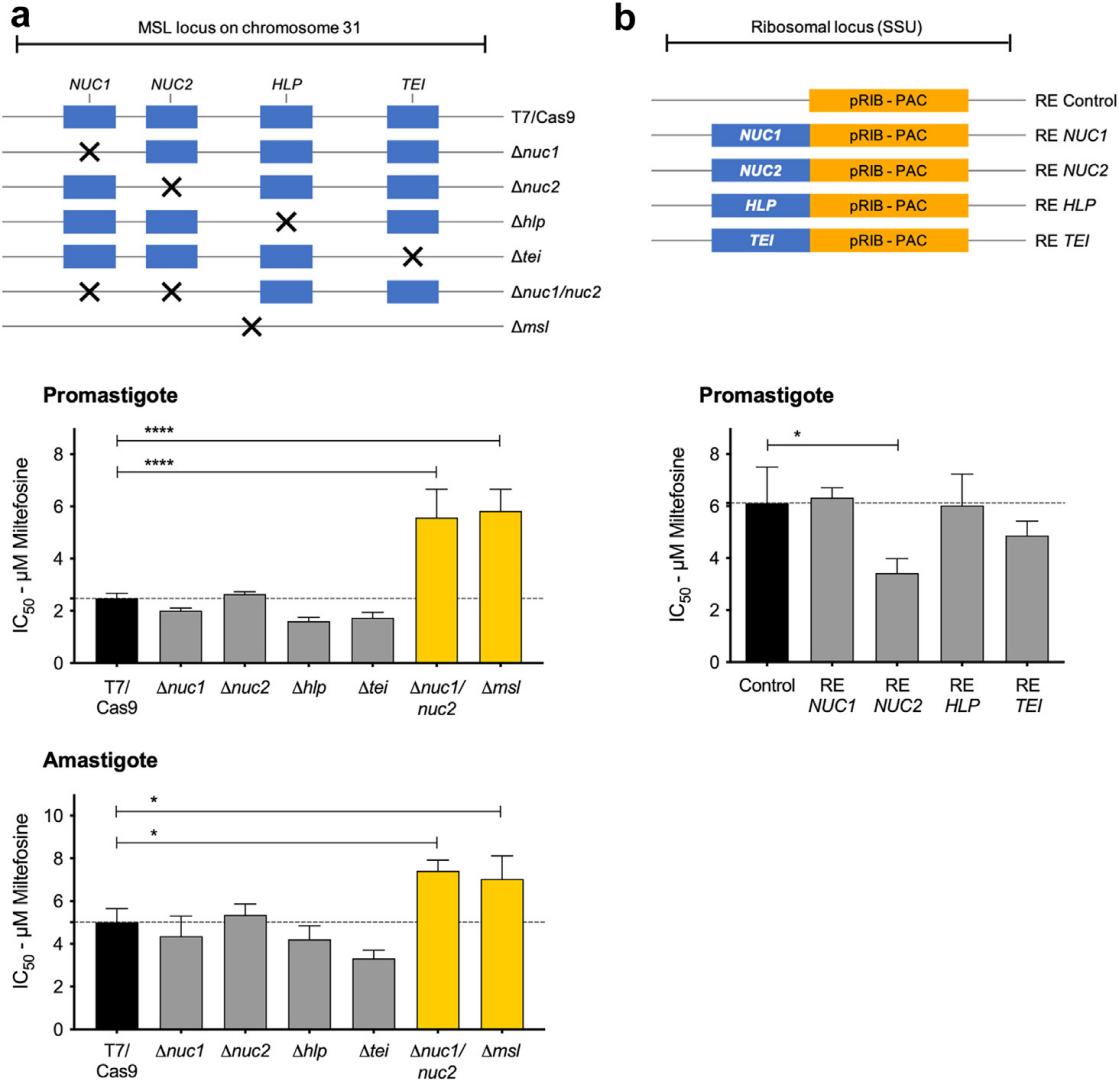
**The 3′-nucleotidase/nuclease is the key component of MSL for the miltefosine susceptibility phenotype in *L. infantum***. MSL genes were deleted using CRISPR Cas9 from an MSL^+^ strain (a) or expressed into ribosomal locus in an MSL^−^ strain (b) according to the schematic representation shown in the top panels. Miltefosine half maximal inhibitory concentration (IC_50_) were measured in promastigote stage by resazurin assay, and in intracellular amastigote stage by percentage of infected macrophage. Data represents mean ± SEM from three independent biological replicates. P values were calculated using unpaired two-tailed Student's t-tests comparing each cell line with the T7/Cas9 parental or control (empty-linearized-pRIB vector) cell line. ∗ p-value <0.05, and ∗∗∗∗ p-value <0.0001, n = 3. *NUC1* (3′-nucleotidase/nucleases), *NUC2* (3′-nucleotidase/nucleases precursor), *HLP* (helicase-like protein), and *TEI* (3,2-trans-enoyl-CoA isomerase).

The null knockout mutants Δ*nuc1*, Δ*nuc2*, Δ*hlp*, Δ*tei*, Δ*nuc1/nuc2*, and Δ*msl* were confirmed by Illumina whole genome sequence, which also was used to screen for CRISPR off-target issues. Reads were mapped using BWA-MEM v.0.7.17-r1188 to the version 59 of the JPCM5 *L. infantum* reference from TritrypDB. Alignments were sorted and converted using samtools v.1.9. Picard tool MarkDuplicates and AddOrReplaceReadGroups v.2.25.5 were used to mark PCR duplicates and add read groups. Mosdepth v.0.2.6 was used to calculate 500bp windows of coverage, the mean coverage across all chromosomes, and to confirm that chromosome 31 was supernumerary. It was also used to calculate gene coverage against annotations from the reference *L. infantum* JPCM version 59. Sample coverage was normalised per chromosome, and the coverage change was calculated from the relative difference between the comparison and reference. 0.5 was used as a cut off, which equates to one copy change. Freebayes v.1.3.6 was used to generate SNP and INDEL calls across all samples. Vcftools v.0.1.16 was used to filter variants with --max-missing 1, --mac 3 and --minQ30 used to remove poor quality calls or missing sites rather than true variation. The intersections between samples were calculated using rtg-tools v.3.12.1 and VCFtools v.0.1.16. SnpEff version 5.1d was used to annotate the predicted effect and severity using a database built from version 59 of the *L. infantum* JPCM from TritrypDB from CDS sequences, gff annotations and the respective genome assembly. The Illumina sequencing data were deposited under the Bioproject accession number PRJNA494801.

### Generation of *L. infantum* cell lines re-expressing MSL genes

The clone 1 from the *L. infantum* isolate MHOM/BR/05/MG11A (MG11A-C1) that lacks the locus MSL, was used as a parental cell line. This isolate: (i) was obtained before the miltefosine treatment from a Brazilian VL patient who relapsed after the treatment; (ii) and showed a resistant *in vitro* phenotype to miltefosine (IC_50_ of 9.7 ± 1.5 μM and 4.9 ± 0.6 μM for amastigote and promastigote forms, respectively). The MSL genes were individually engineered into pRIB construct^37^ to be integrated and expressed from the rRNA locus of MG11A-C1 parasites. The open read frame (ORF) of each MSL gene was amplified by PCR from 100 ng of MA01A-C1 genomic DNA, using Phusion® High-Fidelity DNA polymerase mixture (New England Biolabs) with the specific primer pairs. The PCR products were then sub-cloned into pGEM®-T Easy Vector (Promega) and subsequently cloned into NotI(HF)-digested pRIB plasmid. The integration cassette was finally excised by digestion with *Pac*I and *Pme*I before transfection of MG11A-C1. The empty pRIB vector was used to generate the control cell line. 10^7^ cells of MG11A-C1 in promastigote logarithmic stage were transfected with 5–7 μg of integration cassette, using P3 Primary Cell 4D-Nucleofector (Lonza): one pulse with program FI-115 in a final volume of 110 μL. Electroporated cells were immediately transferred into pre-warmed medium and after 16 h recovery, they were selected and cloned in complete SMD79 solid medium supplemented with 100 μg mL^−1^ of puromycin. Genomic DNA from recovered clones were then extracted and diagnostic PCRs were performed. Primers used to generate the constructs, as well as the ones used for diagnostic PCRs are available in Supplementary Table S1.

### *In vitro* susceptibility to miltefosine

To establish the miltefosine IC_50_ for promastigote form of *L. infantum* cell lines, 5 × 10^5^ parasites mL^−1^ were treated with two-fold increasing concentrations of the drug for 72 h at 25 °C in a 96-well plate. Cell viability was measured by the established colorimetric assay Alamar Blue, which detects metabolically active cells as a summation of viability and proliferation. Subsequently to the drug treatment, 50 μL of 0.0125% (w/v) resazurin (solution prepared in PBS) was added and cells were incubated for additional 2–4 h at 37 °C. Fluorescence emission of the reduced resazurin was detected using a CLARIOstar® reader (BMG LABTECH; excitation filter at 540 nm and emissions filter at 590 nm). The miltefosine susceptibility of intracellular amastigote was performed using Bone Marrow Derived Macrophages (BMDM), which were infected with late-log phase promastigotes at ratio of 10 parasites to 1 macrophage, using the 16 well Labtek tissue culture slides (Nunc, NY, USA). After 24 h of incubation in DMEM supplemented with 5% hi-FBS in 5% CO2 at 37 °C, free promastigotes were removed, and the culture were treated with two-fold increasing concentrations of the drug prepared in DMEM medium supplemented with 2% hi-Horse serum. After 72 h of additional incubation, the slides were stained with Giemsa and 100 cells in each well were counted to determine the percentage of infected macrophages. Fitting of dose–response curves and IC_50_ calculation were carried out using GraphPad Prism v7.0a, considering the untreated control for each cell line as 100% viability.

### Live cell imaging

Promastigote form of the endogenous tagged cell lines in logarithmic phase were washed once at 2000 g for 5 min with PBS, resuspended in 10 μg mL^−1^ of Hoechst 33342 (Thermo Scientific) and then incubated for 15 min at room temperature, protected from light. Subsequently, cells were washed at 2000 g for 5 min with PBS at 4 °C and resuspended in 50 μL of ice-chilled CyGEL™ (Biostatus) to immobilise cells. Cells were transferred to a slide, covered with a coverslip and immediately imaged using a Zeiss LSM 880 with Airyscan on an Axio Observer.Z1 inverted confocal microscope, with 488 and 405 nm lasers, at room temperature. Images were processed using Image J v.2.0.0.

### Protein level by flow cytometry

Promastigote form of the endogenous tagged cell lines, in logarithmic phase, at 5 × 10^5^ cell mL^−1^ were treated with different miltefosine concentrations during multiple time courses at 25 °C. After the treatment, cells were washed once at 2000 g for 5 min with PBS at room temperature and resuspended in 500 μL of PBS. Cells were analysed for FACS using a Beckman Coulter CyAn ADP flow cytometer and Median Fluorescence Intensity (MFI) were measured by FlowJo™ 10.6.2 software. The untagged parenteral T7/Cas9 cell line (P) was used as control to subtract auto fluorescence and protein fold change level was calculated as follow: MFI_(fold change)_ = [(MFI_tagged_treated_) - (MFI_P_treated_)]/[(MFI_tagged_untreated_) - (MFI_P_untreated_)].

### Cell cycle analysis

For cell cycle analysis, promastigote form *L. infantum* isolates were incubated or not for 24 h with miltefosine at different concentrations. Cells were washed once in PBS-EDTA (PBS supplemented with 5 mM of EDTA) and resuspended in 70% methanol. After overnight incubation at 4 °C, cells were washed once with PBS-EDTA and then resuspended in 1 mL PBS-EDTA containing 10 μg mL^−1^ of propidium iodide and 10 μg mL^−1^ of RNase A. Cells were incubated for 45 min at 37 °C in the dark until FACS analysis. Cells were analysed for FACS using AttuneTM NxT Acoustic Focusing Cytometer (v.2.5.391.2) and the data were analysed using FlowJo™ 10.6.2 cell cycle algorithm: Dean-Jett-Fox model.

### Lipid analysis

The lipid metabolites were extracted from cell pellets (4 × 10^7^ cells) of miltefosine-treated and control samples with isopropanol (400 μL) and, 10 μL of deuterated internal standard mix (SPLASH lipidomix, part number: 330707; Avanti Polar Lipids, AL, USA) was included during extraction. Samples were incubated at 95 °C for 15 min and then cooled at 4 °C before 600 μL hexane was added, followed by vortexing and centrifugation (10 min at 18,000 g). The supernatant was transferred to a clean tube, aqueous Na_2_SO_4_ (500 μL, 6.7% w/v) was added, followed by centrifugation to promote phase separation. From the organic layer (top layer), 200 μL was transferred to a glass vial and dried in a centrifugal evaporator. Samples were reconstituted in 200 μL acetonitrile:isopropanol (70:30) and 2 μL injected on LC-MS for analysis of lipids. Three technical replicates were extracted for each sample.

The LC system used was a Waters Acquity UPLC I-Class System (Waters Corporation, USA), fitted with an Accucore C30 column (100 mm x 2.1 mm, 2.6-μm particle size, part number-27826-102130, Thermo Scientific) and an Accucore C30 guard cartridge (10 mm x 2.1 mm, part number-27826-012105, Thermo Scientific). The column temperature was maintained at 40 °C. The weak wash solvent was methanol (5% v/v), and the strong wash solvent was isopropanol. The mobile phase system for elution was: Mobile Phase A-acetonitrile:water (60:40) + 10 mM ammonium formate and formic acid (0.1% v/v), and Mobile Phase B-acetonitrile:isopropanol (10:90) + 10 mM ammonium formate and formic acid (0.1% v/v). Lipids were eluted at a mobile phase flow-rate of 0.35 mL min^−1^, using the following gradient: start at 1% B, followed by a linear increase to 99% B at 21 min, maintain at 99% B until 24 min, return to 1% B at 24.1 min, and this was maintained until 28 min.

Mass spectra were acquired using a Thermo Scientific Orbitrap Fusion Tribrid Mass Spectrometer (Thermo Fisher Scientific, USA) and a heated electrospray ionization (HESI) ion source. The ion transfer tube was set to 300 °C and the HESI vaporizer to 280 °C. Thermo Scientific Xcalibur 4.0 software (Thermo Fisher Scientific, USA) was used to control the UPLC and MS instruments. Alternate injections were made in positive and negative ionisation modes. The spray voltage was 3500 V for positive ionisation mode and 3000 V for negative ionisation mode. Nitrogen gas flows for sheath, auxiliary, and sweep gases were 42, 14, and 1 respectively. MS spectra were acquired over *m*/*z* range of 400–1600. Data analysis was performed using Progenesis® QI software (Waters Corporation, USA). Lipids were identified by searching *m/z* against LipidBlast database^38^ and taking into account retention times and adducts formed by lipid standards (deuterated internal standard mix- SPLASH lipidomix) and mass accuracy (maximum mass error, 5 ppm).

### Miltefosine analysis

Miltefosine was identified and quantified in the samples extracted and processed for lipid analysis by comparison with retention time and peak area of miltefosine external standard (Sigma-Alrdich) run alongside the samples. Miltefosine was detected in positive ionisation mode and *m/z* for [M+H]+ adduct was 408.3238.

### Ergosterol analysis

Ergosterol was extracted from cell pellets (4 × 10^7^ cells) of miltefosine-treated and control samples using the extraction method described for lipid analysis with d_7_-cholesterol included as internal standard (SPLASH lipidomix, p/n 330707; Avanti Polar Lipids, AL, USA). The extract (2 μL) was injected on the LC-MS and analysed using the method described for lipid analysis. Identification and quantification of ergosterol in samples was performed by comparison with retention time and peak area of ergosterol external standard (Sigma–Aldrich) run on the LC-MS alongside the samples. Ergosterol was detected in positive ionisation mode and *m/z* for [M-H2O+H]+ adduct was 379.3352.

### Fatty acid analysis

1 M methanolic HCl (500 mL) (Sigma–Aldrich) and 10 μL of 5 mg mL^−1^ tripentadecanoin (Sigma–Aldrich) as internal standard were added to *L. infantum* cell pellets (1 × 10^8^ cells), followed by transmethylation of fatty acids at 85 °C for 4 h. The fatty acid methyl esters (FAMEs) were partitioned into 200 μL hexane from the aqueous phase by the addition of 0.9% KCl. FAMEs were analysed by GC-FID according to the method described in Larson and Graham (2001).^39^

### Statistical analyses

Data were collected from at least three independent experiments unless otherwise specified. All statistical analysis was performed using GraphPad Prism v7.0a. The appropriate tests were conducted and are detailed in the corresponding figure legends. All results are expressed by mean ± standard error of mean (SEM).

### Role of the funding source

The funders had no role in the study design, data collection, data analysis, interpretation, writing of the manuscript, or the decision to submit the paper for publication.

## Results

It has been previously shown that miltefosine resistant *Leishmania* lines generated in the laboratory have changes in expression of the miltefosine transporter or its β-subunit Ros3.^12^, ^13^, ^14^ To assess if these same traits are found in the *L. infantum* clinical isolates we carried out a relative quantification of these two genes by qPCR, as well as checked the whole genome sequence for SNPs/InDels and gene dose. These analyses were performed in 26 *L. infantum* clinical isolates obtained from a controlled trial to evaluated miltefosine efficacy against visceral leishmaniasis in Brazil,^32^ which included 14 isolates from patients who showed cure after the six months follow up, and 12 isolates from patients who relapsed during the follow up. In this previous study, we showed a significant correlation between clinical outcome and *in vitro* susceptibility to miltefosine. However, no significant difference in gene expression of the miltefosine transporter and its β-Ros3 subunit were observed in these *L. infantum* clinical isolates (Fig. 1a and b) and no SNPs or InDels were found in those genes (SRA project ID PRJNA494801, with the following weblink https://www.ncbi.nlm.nih.gov/sra/?term=PRJNA494801). We also measured the accumulation of miltefosine in six of the *L. infantum* isolates and no difference between the cured and relapsed groups was detected after 3.5 h or 5.0 h of drug pressure (Fig. 1c).

We also checked the ability of these *L. infantum* field isolates to control miltefosine-induced ROS. To set the best miltefosine treatment conditions in terms of time and concentration, we analysed the kinetics of ROS accumulation under different miltefosine treatments in the reference *L. infantum* strain MHOM/BR/74/PP75 (IC_50_ of 3.09 ± 0.77 μM and 7.74 ± 1.13 μM for amastigote and promastigote forms after 72 h treatment, respectively). A dose and time dependent response was observed at concentrations greater than 10 μM of miltefosine (Supplementary Fig. S2a). It was also observed that the increase of ROS accumulation corresponded with a decrease in parasite viability (Supplementary Fig. S2b). However, no significant difference in the ability to control redox homeostasis was observed between the groups of *L. infantum* isolates from patients who showed different clinical outcomes (Fig. 2).

As we had previously associated miltefosine treatment failure with deletion of MSL locus on chromosome 31 of *L. infantum*,^33^ we next investigated the role of MSL locus in the mechanism of miltefosine resistance. The MSL contains four genes: 3′-nucleotidase/nucleases LINF_310031200 (*NUC1*); 3′-nucleotidase/nucleases precursor LINF_310031300 (*NUC2*); helicase-like protein LINF_310031400 (*HLP*); and 3,2-trans-enoyl-CoA isomerase LINF_310031500 (*TEI*). To be able to manipulate the *L. infantum* parasite genome we first engineered a clonal cell line MSL^+^ (MA01A-C1), which had *in vitro* susceptibility to miltefosine, to express the T7 RNA polymerase and Cas9 endonuclease. Then, using CRISPR Cas9 technology we generated knockout cell lines for each gene in the MSL individually, Δ*nuc1* (*NUC1*), Δ*nuc2* (*NUC2*), Δ*hlp* (*HLP*), and Δ*tei* (*TEI*), for both 3′-nucleotidase/nucleases, Δ*nuc1/nuc2*, and for the whole MSL locus Δ*msl* (Supplementary Fig. S3 and Fig. 3a). The whole genome sequence analysis confirmed the specific gene/locus deletions in all the MSL knockout mutants. The absence of reads covering the MSL gene/locus across the genome of *L. infantum*, also excludes the occurrence of translocation of the target gene/locus to another genomic locus (Supplementary Fig. S4).

Although the nucleotide sequence of the sgRNA and the PAM drives Cas9 nuclease cleavage specificity, off-target events have been reported in CRISPR-Cas system.^40^^,^^41^ In eukaryotic cells, this off-target DNA break is repaired by classical non-homologous end-joining (c-NHEJ), entailing small deletions or insertions. However, in the trypanosomatids a functional c-NHEJ pathway is lacking,^42^ and DNA end-joining is repaired using regions of micro-homology (MMEJ) or single-strand annealing (SSA), which are frequently associated with deletion.^43^^,^^44^ In this context, it is unlikely that cleavage at off-targets sites, for which no repair template was provided, will select parasite with unintended editing. Nevertheless, to assess the potential occurrence of Cas9-induced off-target editing, coverage of all genes was measured, and insertions and deletions (InDels) searched throughout the genome sequence of the knockout lines. Differences in read depth coverage (RDC) between knockout lines and T7/Cas9 showed that the MSL genes were the only ones completely deleted. However, gene copy differences, most of which (98.38% in average) had RDC differences ranging from 0 to 0.3, were observed, indicating minimal fluctuations. Besides, RDC difference higher than 0.5 were predominantly observed in multicopy genes and/or genes with multiple paralogues, which are prone to mapping errors due to short reads from Illumina sequencing. The other coding sequences (CDS) that showed RDC difference >0.5, are likely to be natural fluctuation, since the RDC of mutants are similar to MA01A, the parental line of T7/Cas9 (Supplementary Fig. S5 and Supplementary Table S2). These variations are probably derived from *Leishmania* genome plasticity and are unlikely to have resulted in a mutant-specific phenotype. Small insertion and deletions throughout the whole genome of the transgenic lines were also identified when compared with T7/Cas9 genome. However, most of these variations were found in the repetitive sequences of intergenic regions, suggesting that they are false positive due to low sequence complexity. After filtering out variants shared between the mutants and T7/Cas9 and/or its parental line MA01A, no new InDels (present in the mutant(s), but not in T7/Cas9 or MA01A) were identified on coding sequences of the MSL knockout lines, suggesting the observed genotype fluctuations is due to natural variations (Supplementary Table S3). Taken together, these results indicate that no significant off-target variations were caused by genetic engineering using CRISPR/Cas9 system.

Δ*nuc1/nuc2 and* Δ*msl* mutants displayed significantly reduced miltefosine susceptibility in promastigote and amastigote forms, whereas Δ*nuc1*, Δ*nuc2*, Δ*hlp*, or Δ*tei* mutants did not (Fig. 3a), pinpointing the 3′-nucleotidase/nuclease as the key component of the MSL involved in miltefosine susceptibility. Furthermore, expression of *NUC2,* but not *NUC1*, *HLP* or *TEI* from the ribosomal locus of a clonal cell line lacking the MSL (MG11A-C1) led to a significant increase in the *in vitro* miltefosine susceptibility in the promastigote stage (Fig. 3b).

**Fig. 4:**
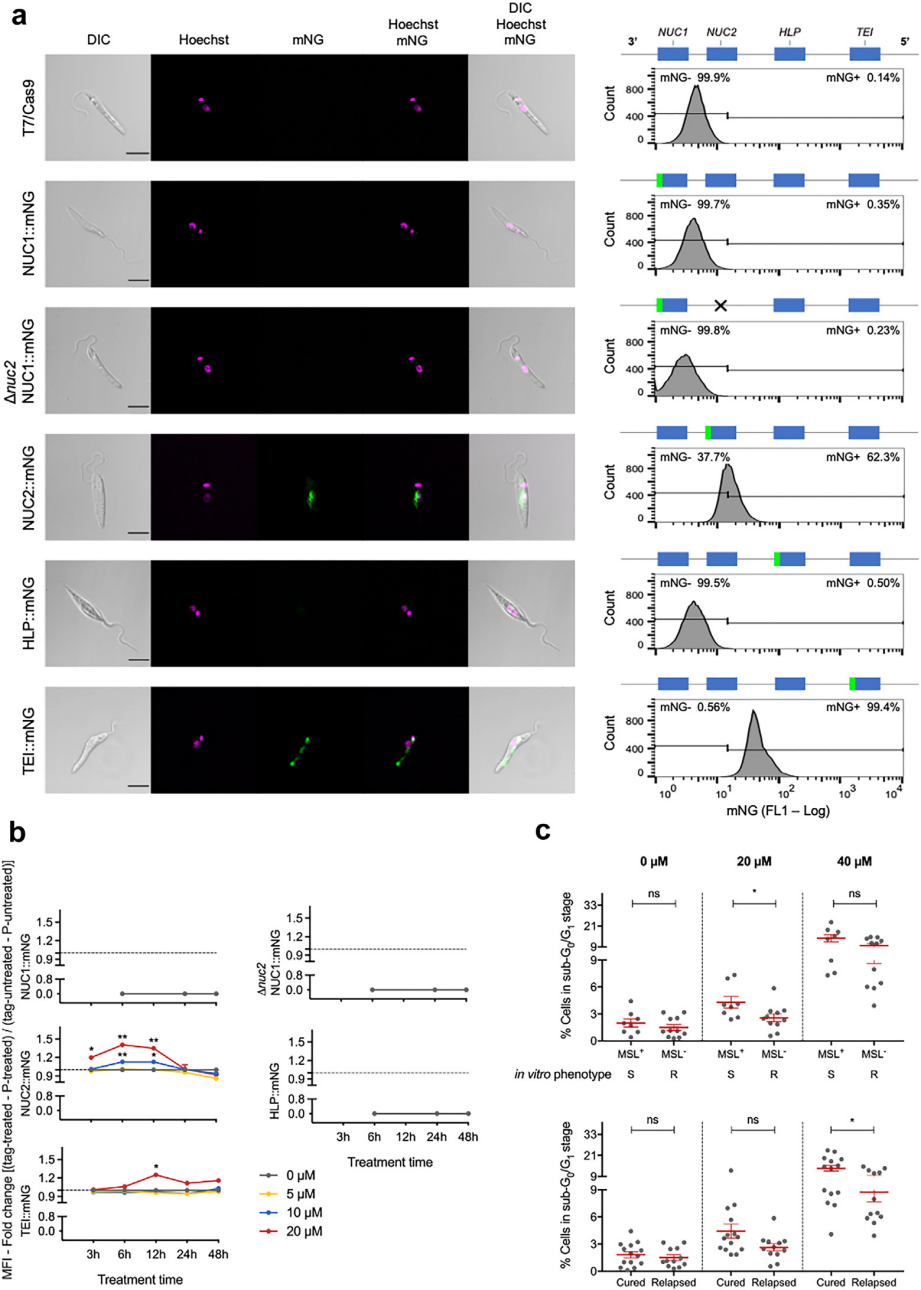
**MSL proteins localisation and expression level**. (a) Representative fluorescence microscopy micrographs, showing promastigote form of *L. infantum* endogenously expressing C-terminal mNeonGreen (mNG) tagged MSL proteins. Cells were counterstained with Hoechst 33342 to visualise DNA. The right panel shows a representative histogram of mNG fluorescence detection by flow cytometry. (b) Relative expression of mNG tagged MSL protein during miltefosine treatment. Data represents mean ± SEM from three independent biological replicates. P values were calculated using two-tailed Student's t-tests comparing the treated with untreated for each time point (∗ p-value <0.05; ∗∗ p-value <0.01). (c) Level of DNA degradation evaluated through cell cycle analysis of cells containing sub-G_0_/G_1_ DNA content. Each grey dot represents the mean of individual *L. infantum* isolates from three independent experiments. Mean and ±SEM for cured and relapsed or MSL^+^/S and MSL^−^/R groups are shown in red. P values were calculated using unpaired two-tailed Student's t-tests comparing the group cured with relapsed or MSL^+^ with MSL^−^ (ns, not significant; ∗ p-value <0.05). S, miltefosine sensitive *in vitro* phenotype; R, miltefosine resistant *in vitro* phenotype. Gene (product description): *NUC1* (3′-nucleotidase/nucleases), *NUC2* (3′-nucleotidase/nucleases precursor), *HLP* (helicase-like protein), and *TEI* (3,2-trans-enoyl-CoA isomerase).

Using CRISPR Cas9 we also generated cell lines to express the MSL proteins endogenously tagged with mNeonGreen (mNG) at the C-terminus. Both alleles were tagged. The cellular localisation and protein expression of the MSL proteins was then explored by fluorescent microscopy and flow cytometry, respectively (Fig. 4). NUC2:mNG, was found primarily in the perinuclear area of the parasite and its expression was detected in about 60% of the cells. TEI:mNG was localised to the mitochondrion with a focus at the kinetoplast. No protein expression was detected for NUC1:mNG or HLP:mNG (Fig. 4a).

In addition, we also checked if miltefosine can modulate the expression/stability of the MSL proteins, as a direct or indirect effect of miltefosine perturbation in the cell, which can assist in uncovering the mode of action of the drug. The effect of miltefosine on the level of the C-terminal mNG fusion proteins was followed by flow cytometry. Miltefosine did not change the protein level of NUC1:mNG or HLP:mNG, which remained without detectable expression. However, a concentration higher than 10 μM miltefosine increased the protein level of NUC2:mNG between 3 and 6 h after exposure, and its expression level returned to baseline level after 24 h. Miltefosine also changed the expression level of TEI:mNG after 6 h treatment, which reached maximum level at 12 h and returned to basal level in 24 h (Fig. 4b).

Considering that the NUC2 protein is predicted to act in DNA catabolic processes, and that miltefosine increased the protein level of this enzyme, we checked if *L. infantum* isolates from cured patients are more susceptible to DNA degradation, under miltefosine pressure, than isolates from relapsed patients. DNA degradation was evaluated through cell cycle analysis of cells containing sub-G_0_/G_1_ DNA content.^45^ A significant difference between cured and relapsed groups was observed at 40 μM miltefosine after 24 h treatment. Furthermore, a significant difference was also observed when the isolates were sorted by the presence of the MSL locus and *in vitro* miltefosine phenotype (Fig. 4c and Supplementary Fig. S6).

**Fig. 5:**
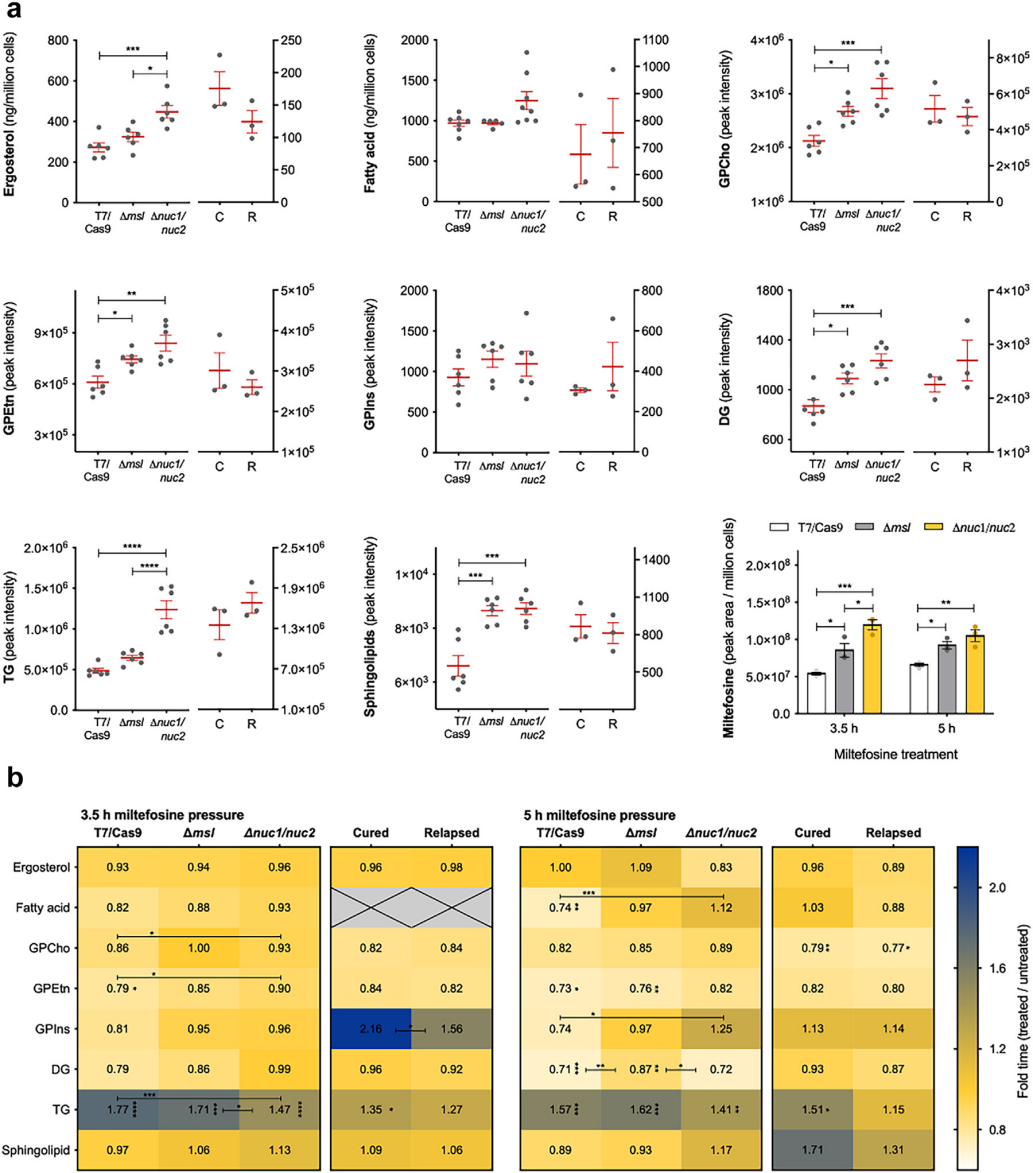
**Lipidomic analysis of *L. infantum***. The lipidomic analysis were carried out with parental T7/Cas9, Δ*msl* and Δ*nuc1/nuc2*, and 6 *L. infantum* isolates (3 from cured (C)/MSL^+^/sensitive *in vitro* and 3 from relapsed (R)/MSL^−^/resistant *in vitro* groups) in logarithmic promastigote stage. (a) Lipid content baseline of the *L. infantum* cell lines and clinical isolates and miltefosine accumulation in the parasite. Each grey dot represents an independent experiment (n = 6) for the isogenic cell lines or the mean of an individual *L. infantum* isolate from three independent experiments. Miltefosine level was measured after 3.5 h and 5 h treatment with 10 μM of the drug. Mean and ±SEM are shown in red. P values were calculated using one-way ANOVA comparing the cell lines; and using unpaired two-tailed Student's t-tests comparing the group cured with relapsed. (b) Heat map showing the effect of 10 μM of miltefosine on the parasite lipid content. P values were calculated using unpaired two-tailed Student's t-tests comparing: the pairs of untreated with treated, to check if miltefosine treatment is causing a significant perturbation in lipid content (asterisk next to the ratio value in each cell); and the lipid variation caused per miltefosine treatment between the groups of cell lines or clinical isolates, to check if some group is significant more susceptible/resistant to lipid perturbation caused by miltefosine (asterisk over the bar). GPCho, glycerophosphatidylcholines; GPEtn, glycerophosphatidylethanolamines; GPIns, glycerophosphatidylinositols; DG, diacylglycerol; TG, triacylglycerol. ∗ p-value <0.05; ∗∗ p-value <0.01; ∗∗∗ p-value < 0.001; ∗∗∗∗ p-value < 0.0001.

Miltefosine is a phospholipid analogue and has been shown to cause perturbation of lipid membranes and lipid metabolism in *Leishmania* parasites. We therefore performed a metabolomic analysis of the parasite lipid content (fatty acids, glycerolipids, glycerophospholipids, sphingolipids and sterol) on the parental T7/Cas9, Δ*msl* and Δ*nuc1/nuc2* cell lines, together with three *L. infantum* MSL^+^ isolates and three MSL^−^ isolates. The lipid content baseline did not show significant difference between MSL^+^ and MSL^−^ *L. infantum* isolates. However, the resistant isogenic cell lines (Δ*msl* and Δ*nuc1/nuc2*) displayed a significant higher baseline content of glycerophosphatidylcholines (GPCho), glycerophosphatidylethanolamines (GPEtn), diacylglycerol (DG) and sphingolipids compared with the T7/Cas9. The ergosterol and triacylglycerol (TG) baseline content were significantly higher in Δ*nuc1/nuc2* than in T7/Cas9, with Δ*msl* showing an intermediate content. No difference was observed in the baseline level of fatty acid and glycerophosphatidylinositols (GPIns) between the isogenic cell lines (Fig. 5a). Surprisingly, the resistant isogenic cell lines showed a substantially higher miltefosine accumulation level compared to T7/Cas9, suggesting that miltefosine level was inversely associated with the drug susceptibility (Fig. 5a).

Miltefosine treatment triggered several alterations in lipid content of *L. infantum* parasites, such as a general decrease in fatty acid, GPCho, GPEtn and DG lipid content and increase of TG level. The levels of ergosterol and sphingolipid were not altered by miltefosine (Fig. 5b). It was also notable that fatty acid, and GPEtn were not disturbed in the Δ*nuc1/nuc2* mutant. Furthermore, Δ*nuc1/nuc2* and the isolates from the relapsed group exhibited better control of TG content under miltefosine pressure (Fig. 5b). Altogether, the lipidomic analysis indicates that miltefosine resistant *L. infantum* parasites are also more resistant to lipid content perturbation caused by miltefosine and that MSL, or more specifically the 3'nucleotidase/nuclease, plays a direct or indirect role in the control of lipid metabolism.

**Fig. 6:**
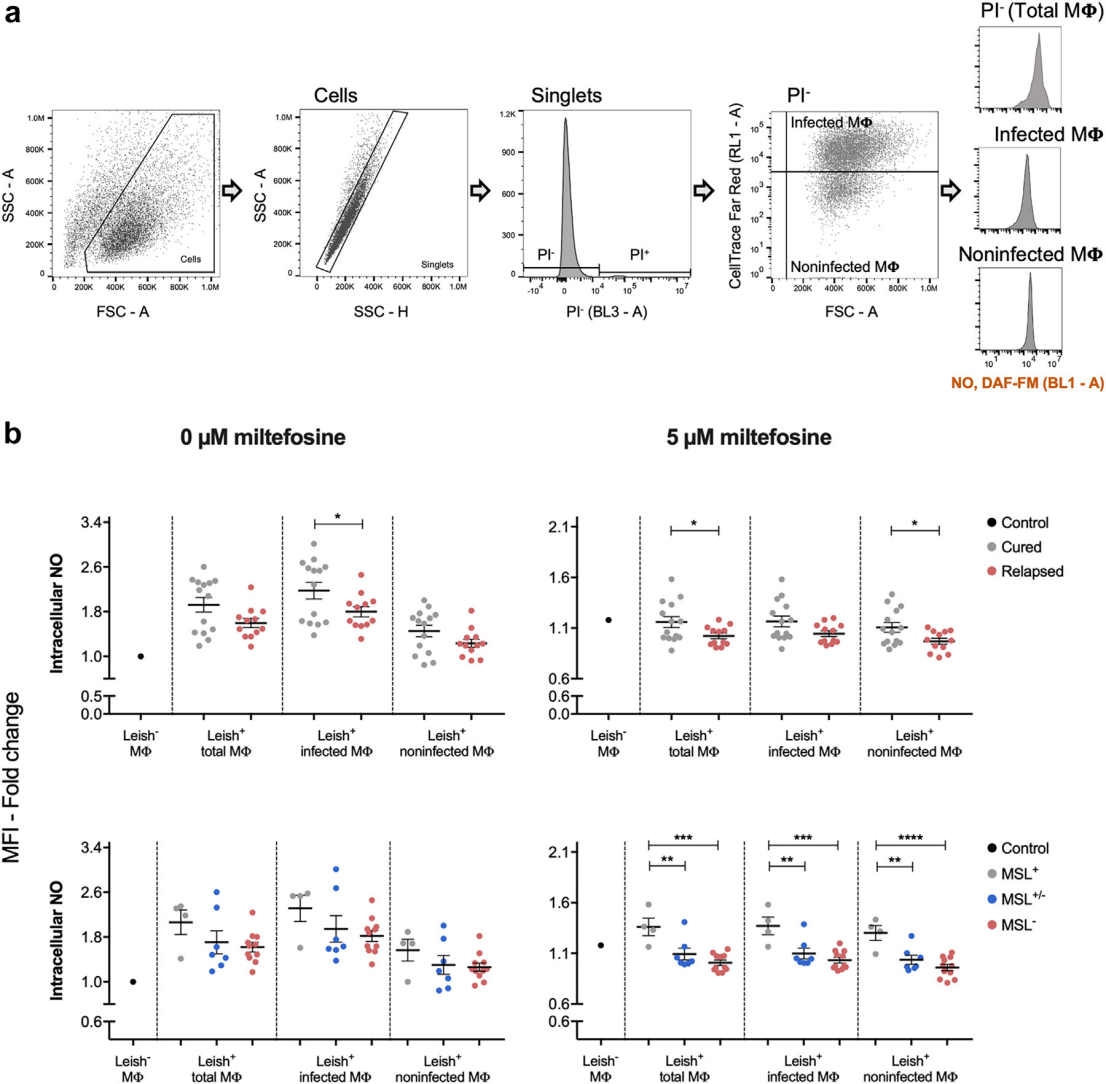
**Intracellular level of NO in RAW264.7 cells infected with different *L. infantum* isolates**. RAW264.7 cells incubated for 24 h with *L. infantum* (26 isolates: 14 from cured group and 12 from relapsed group. The isolates were also sorted by their MSL genotype/clinical outcome: 4 MSL^+^/cured, 7 MSL^+/−^/cured, and 11 MSL^−^/relapsed) were subsequently treated with 0 or 5 μM of miltefosine for an additional 24 h. (a) Gate strategy used to analyse the intracellular level of NO into different populations. (b) The intracellular level of NO, in RAW264.7 cells was measured using DAF-FM (Invitrogem™). In the absence of miltefosine, the cells were normalised by the untreated not challenged RAW264.7 cells (Control Leish^−^ MΦ), whereas the miltefosine treated RAW264.7 cells (infected or not with *L. infantum* parasite) were normalised by their untreated pair. Each dot represents the mean of individual *L. infantum* isolates from three independent experiments. Mean and ±SEM for the groups is shown in black. P values were calculated using unpaired two-tailed Student's t-tests comparing the group cured with relapsed; and using One-way ANOVA test comparing the groups MSL^+^, MSL^+/−^ and MSL^−^ (∗ p-value <0.05; ∗∗ p-value <0.01; ∗∗∗ p-value <0.001; ∗∗∗∗ p-value <0.0001). MΦ, macrophage; Leish^−^, not infected with *L. infantum* parasite; Leish^+^, infected with *L. infantum* parasite.

Furthermore, it is also known from our previous study^33^ that some *L. infantum* isolates from cured patients comprise a mixed population of MSL^+^ and MSL^−^ parasites. In an attempt to better understand the contribution of the different genotypes, we investigated the ability of *L. infantum* MSL^+^, MSL^+/−^ and MSL^−^ isolates to control the redox environment inside of the macrophage cell line RAW264.7. We first demonstrated that this macrophage cell line can be simultaneously infected with isolate MSL^+^ and MSL^−^, staining each isolate with different CellTrace™ (Supplementary Fig. S7). It was also observed that, in the absence of miltefosine, the accumulation of nitric oxide (NO), mitochondrial superoxide (•O_2_^-^) and ROS (H_2_O_2_, HO•, ROO• and ONOO^−^) in macrophages was generally higher in the system challenged with any *L. infantum* isolate (Leish^+^ MΦ) than in the control system not challenged with the parasite (Leish^−^ MΦ). This increase was also observed in the majority of noninfected-macrophage population from the system challenged with *L. infantum* parasite, indicating that noninfected macrophages were also modulated by *Leishmania* infection (Fig. 6 and Supplementary Fig. S8). A significantly higher NO accumulation, in the infected macrophage population, was observed in the group of *L. infantum* isolates from cured patients. However, the role of MSL on this modulation was not clear since a gradual decrease in NO accumulation was observed in MSL^+^, MSL^+/−^ and MSL^−^ strains, but no significant difference was detected (Fig. 6). In the absence of miltefosine, the level of •O_2_^-^ accumulation through RAW cell populations was also different between the MSL^+^ and MSL^+/−^ isolates and MSL^−^ and MSL^+^ isolates (Supplementary Fig. S8).

When miltefosine was added to the cultures, the ability of the parasites from relapsed patients to favourably control the intracellular level of NO in RAW cells was significantly higher than in the cured group. This difference was observed in the total macrophage and in the noninfected macrophage populations, suggesting the importance of the whole environment to the control of *Leishmania* infection (Fig. 6). A gradual NO accumulation level through the isolates MSL^+^, MSL^+/−^ and MSL^−^ with significant difference only between MSL^+^ and MSL^+/−^ and between MSL^+^ and MSL^−^ was also observed (Fig. 6).

## Discussion

It has been documented that the effectiveness of miltefosine against VL has declined over time since its introduction in India in 2002.^9^, ^10^, ^11^ The failure of miltefosine to treat VL caused by *L. donovani* has been mainly associated with an increase in parasite fitness^46^ than with drug susceptibility.^9^^,^^47^ On the other hand, we showed previously that miltefosine treatment failure in Brazilian VL, caused by *L. infantum*, correlated with natural-miltefosine resistance, which in turn was associated with deletion of the MSL locus.^32^^,^^33^ In this present study we show that these Brazilian *L. infantum* isolates, from patients with different treatment outcomes, did not differ in their ability to control drug-induced oxidative stress in the promastigote stage or in their miltefosine uptake machinery, as previous revealed for experimentally selected miltefosine-resistant *Leishmania* parasites.^12^, ^13^, ^14^, ^15^, ^16^^,^^25^^,^^29^ It has been shown that *L. infantum* parasites resistant to miltefosine because of deficient miltefosine transport have a profound loss of fitness.^48^ The same research group also found that although a natural miltefosine resistant strain deficient in the β-subunit Ros3 did not develop mature infections and metacyclogenesis in the sand fly vector, it was significantly hampered in its ability to multiply and cause a standard visceral infection in BALB/c mice,^49^ explaining most likely the absence of this widespread miltefosine resistance in *L. infantum* in the field.

In this study we focused our analysis on the role of MSL in the miltefosine resistance mechanism, since its deletion was previously correlated with miltefosine treatment failure and *in vitro* drug resistance in *L. infantum*.^32^^,^^33^ The data presented here confirmed that MSL deletion is associated with *in vitro* miltefosine resistance and that the two 3'nucleotidase/nucleases NUC1 and NUC2 are the key component of the MSL that plays a role in the miltefosine susceptibility phenotype. The deletion of both NUC1 and NUC2 from the endogenous locus was required to alter susceptibility to miltefosine, whereas the re-expression of NUC2 only, from the ribosomal locus, changed the phenotype. This variation could be caused by natural differences between the two parental lines used to delete the MSL (MA01A T7/Cas9 that naturally contains the MSL) or re-express MSL genes (MG11A that naturally lack MSL) (Supplementary Fig. S9, Supplementary Tables S2 and S4, and Carnielli et al.^33^). An additional factor is that re-expression of the MSL genes were done from the ribosomal locus, rather than their endogenous locus.

The 3'nucleotidase/nuclease is a bifunctional enzyme, member of the class I nuclease family, that can hydrolyse both 3′-monophosphorylated nucleotides and nucleic acids. NUC1 shares 63% amino acid sequence identity with NUC2, which has orthologs in all *Leishmania* species with their genomes sequenced. On the other hand, no orthologs for NUC1 were found in *Leishmania amazonensis*, *Leishmania enriettii*, *Leishmania mexicana*, and *Leishmania tarentolae*. Moreover, a transmembrane domain ending 20 and 18 amino acids from the C-terminus is predicted for NUC1 and NUC2, respectively. NUC2 also has a signal peptide predicted at its N-terminus (Supplementary Fig. S10). Although our data indicate a perinuclear localisation for the *L. infantum* NUC2, its ortholog in *L. donovani* has been characterised as a cell surface membrane-anchored protein with a N-terminal signal peptide that targets the enzyme to the endoplasmic reticulum and a C-terminal transmembrane domain that anchors the enzyme to the parasite surface.^50^^,^^51^ On the other hand, the *Leishmania pifanoi* P-4 nuclease, an amastigote stage specific protein that shared a 34% identity with the *L. donovani* 3'nucleotidase/nuclease, was found to localise to the nucleus/perinucleus, suggesting that P-4 nuclease potentially may be involved in RNA stability (gene expression) and/or DNA excision/repair.^52^

As a purine auxotroph, *Leishmania* are strictly dependent on the purine salvage process to acquire this essential nutrient (reviewed^53^), and the 3'nucleotidase/nuclease has been pinpointed as a key enzyme in this pathway.^54^^,^^55^ Furthermore, it has been observed that 3′nucleotidase/nuclease is also important to allow *Leishmania* parasites to escape killing by neutrophil extracellular traps^56^ and to favour parasitic infection in host macrophage.^57^^,^^58^ However, no difference in the ability to infect macrophage by *L. infantum* MSL^+^ and MSL^−^ was observed in our previous study.^32^ It is noteworthy that on *L. infantum* chromosome 31 two ORFs coding for the 3'nucleotidase/nuclease are predicted, but only the fused NUC2:mNG protein, displayed a detectable expression level in the promastigote stage in our study. Also, *L. infantum* parasite has another 3'nucleotidase/nuclease paralog on chromosome 12 (LINF_120009100, which a SignalP and a transmembrane domain at N- and C-terminus, respectively, are predicted); and on chromosome 30 (LINF_300020200, which a SignalP at N-terminus is predicted). Protein sequence analysis showed that NUC1 shares 45.7% and 38.6% identity with LINF_120009100 and LINF_300020200, respectively, whereas NUC2 are 43.3% and 35.2% identical to LINF_120009100 and LINF_300020200, respectively (Supplementary Figs. S10 and S11). So, the MSL^−^ *L. infantum* parasites described in our study have other 3'nucleotidase/nucleases that may compensate for loss of *NUC1* and *NUC2*. We also showed that miltefosine pressure increases NUC2:mNG protein level in *L. infantum* promastigotes, which could be linked with the lower resistance to miltefosine-induced DNA degradation, a known type of cell death caused by miltefosine,^59^^,^^60^ observed on MSL^+^ parasites. However, further investigations are required to understand if this increase in protein expression is a result of a direct or indirect effect of miltefosine. Similarly, it is also necessary to investigate if NUC2 is directly involved in DNA degradation or if it is regulating a functional interactor.

Consistent with previous reports^24^^,^^61^ we also found that miltefosine triggers a reduction in phospholipids in *L. infantum* promastigotes. In addition, we also observed a decline in fatty acids and diacylglycerol and an increase in triacylglycerols as an effect of miltefosine exposure. On the other hand, we showed that ergosterol and most sphingolipid levels remained unchanged after exposure to miltefosine, although it has been reported that miltefosine causes an increase in the levels of these lipids.^24^^,^^61^ Besides that, we showed here that the deletion of the MSL or *NUC1/NUC2* were followed by a general increase in baseline lipid content, including ergosterol (1.2 and 1.6 fold change increase in Δ*msl* and Δ*nuc1/nuc2*, respectively), depletion of which has been associated with miltefosine resistance in *L. donovani*.^62^ Taking into account this higher level of ergosterol and the strong affinity of this sterol for miltefosine,^63^ the higher level of miltefosine observed in these resistant cell lines is possibly due to the role of ergosterol as a miltefosine-binding reservoir in the membrane. Matching our results, a study that investigated miltefosine interaction with different lipid membranes suggests that miltefosine molecules incorporated into sterol-rich (cholesterol and ergosterol) membranes will remain kinetically trapped, since this interaction exhibited a very slow desorption rate.^64^

Our lipidomic study also showed that miltefosine resistance due to deletion of the MSL or both NUC1 and NUC2 matched with a better ability of these isogenic cell lines to control the lipidome perturbation caused by miltefosine. Although MSL contains the TEI gene, which codes for an enzyme involved in fatty acid β-oxidation, it seems that the MSL component playing a role in lipid metabolism control is the NUC1 and NUC2 since comparable lipid profiles and miltefosine resistance profiles were observed between Δ*msl* and Δ*nuc1/nuc2* mutants. It is noteworthy that an *in-silico* analysis revealed that all the 3'nucleotidase/nucleases from *L. infantum* contain a phospholipase C domain (Supplementary Fig. S10), the absence of which could possibly cause the lipid content adaptation in the Δ*msl* and Δ*nuc1/nuc2* mutants. Phospholipase activity has been described in mammalian ecto-nucleotidases, some of which have choline head group lipids as substrate, and are involved in multiple biological processes such as cell migration, lymphocyte trafficking, angiogenesis and inflammation (reviewed by Zimmermann & Zebisch 2012^65^). However, further genetic and biochemical investigations are required to verify if the 3'nucleotidase/nucleases have phospholipase activities in *L. infantum*.

Additionally, under miltefosine pressure, the *L. infantum* clinical isolates from relapsed patients showed a better ability to control triacylglycerol variation. It is relevant to mention that, different from the MSL knockout lines, clinical isolates are not isogenic, and it is possible that a multifactorial traits balance results in their phenotype, which could partially explain the lower difference observed in the lipidomic analysis over them.

Given that miltefosine not only induces direct parasite killing but also modulates the mammalian host immunity driving a Th1 response to control *Leishmania* infection (reviewed by Palic et al.^66^), we additionally checked if *L. infantum* isolates from VL patients with different clinical outcome otherwise modulate the redox homeostasis in the host macrophage. Our results showed that *L. infantum* isolates from VL patients who relapsed after miltefosine treatment favourably control NO accumulation in the macrophage, which is the main mechanism known to kill *Leishmania* parasite. An intermediate trend was also observed when these isolates were arranged by MSL presence, suggesting one more possible role of MSL in *L. infantum* miltefosine susceptibility phenotype. However, further studies in a complete host immune system (*in vivo* model), using isogenic parasites, are necessary to establish the function of MSL on parasite survival into the host. These studies will also bring some lights to the knowledge if the magnitude of NO modulation caused by MSL mixed population (MSL^+/−^) isolates is enough to alter the microenvironment of macrophages toward leishmanicidal type, resulting in parasite elimination and cure of the patients after miltefosine treatment, addressing the paradoxical context where VL patients infected with MSL^+/−^ isolate cure after miltefosine treatment.

Understanding miltefosine resistance in *Leishmania* is important for preserving its clinical use and also to rationally provide multidrug therapy combinations of miltefosine with standard and/or new anti-leishmanial drugs. In this work we established the involvement of MSL in *L. infantum* miltefosine susceptibility and highlight the 3'nucleotidase/nuclease as being a key enzyme.

## Contributors

Conceived and designed the experiments: JCM, JBTC, AD, AR, IAG. Performed experiments: JBTC, AD, PRF, RMR. Analysed data: JBTC, AD, AR, SF, RMR, IAG, JCM. Gathered the clinical sample and data: CHNC, RD. Verified the data: JBTC, AD, AR, SF, JCM. Wrote the manuscript: JBTC, JCM. All authors read and approved the final manuscript.

## Data sharing statement

All raw DNA-sequencing data generated by this study can be accessed through SRA project ID PRJNA494801, with the following weblink https://www.ncbi.nlm.nih.gov/sra/?term=PRJNA494801. Table with all CDS normalized was deposited on Figshare, DOI: https://doi.org/10.6084/m9.figshare.21318240. Table with all variants identified was deposited on Figshare, DOI: https://doi.org/10.6084/m9.figshare.21334602. Additional data generated by this study are available as supplementary information and from the corresponding author on reasonable request.

## Declaration of interests

The authors declare no competing interests.
