# Supplementary material for: 3′Nucleotidase/nuclease is required for Leishmania infantum clinical isolate susceptibility to miltefosine
Source: eBioMedicine. 2022 Nov 30;86:104378. doi: 10.1016/j.ebiom.2022.104378 (PMC9713291; doi:10.1016/j.ebiom.2022.104378)
Supplement: Supplementary file 1 — Supplementary Figs. S1–S11 and Tables S1–S4 [file mmc1.pdf]

SUPPLEMENTARY MATERIAL

3'Nucleotidase/nuclease is required for *Leishmania infantum* clinical isolate susceptibility to miltefosine

Juliana B.T. Carnielli D.Sc., Anuja Dave PhD, Audrey Romano PhD, Sarah Forrester PhD, Pedro R. de Faria Msc, Renata Monti-Rocha D.Sc., Carlos H. N. Costa D.Sc., Reynaldo Dietze M.D., Ian A. Graham PhD, Jeremy C. Mottram PhD.

CONTENTS

Supplementary Table 1: Sequence of oligonucleotides. 1

Supplementary Table 2: Read depth coverage differences between MSL mutants/MG11A and *L. infantum* T7/Cas9. 3

Supplementary Table 3: Summary of variants identified in coding sequence region of the *L. infantum* knockout mutants against T7/Cas9. 7

Supplementary Table 4: Summary of variants identified in coding sequence region of the *L. infantum* MG11A against T7/Cas9. 12

Supplementary Figure 1: Map of the plasmid pTB010. 13

Supplementary Figure 2: Concentration and time effect of miltefosine in ROS accumulation and cell viability on promastigote stage of *L. infantum* parasite. 14

Supplementary Figure 3: PCR screening of the cell lines generated. 15

Supplementary Figure 4: Whole Genome Sequence analysis of *L. infantum* isolates/mutants. 17

Supplementary Figure 5: Read depth coverage differences between MSL mutants and *L. infantum* T7/Cas9. 18

Supplementary Figure 6: Effect of miltefosine on the cell cycle of *L. infantum* promastigotes. 19

Supplementary Figure 7: Multiple *Leishmania* infection in RAW264.7 macrophage. 20

Supplementary Figure 8: Intracellular level of ROS into RAW264.7 cells challenged with different *L. infantum* isolates. 22

Supplementary Figure 9: Read depth coverage differences between *L. infantum* MG11A and T7/Cas9. 23

Supplementary Figure 10: In-silico protein domain analysis for *L. infantum* 3'nucleotidase/nuclease. 24

Supplementary Figure 11: *Leishmania infantum* 3'Nucleotidase/Nuclease protein sequences Analysis. 25

Supplementary Table 1: Sequence of oligonucleotides.

OL ID	Target	Oligonucleotide sequence (5' » 3')	Description
Fw, MT	MT(LINF_130020800)	CAAGTGCCTTTCCACCAGAATC	qPCR, product size of 206 bp
Rw, MT		CTCACCTTTTTGAACTCCAACAGG	
Fw, β-Ros3	β-Ros3 (LINJ_320010400)	ACGACACGGCTTGATTTTCG	qPCR, product size of 228 bp
Rw, β-Ros3		GAGTAGTCCACGGAGGCAGTAAAG	
Fw, GAPDH	GAPDH	GAAGTACACGGTGGAGGCTG	qPCR, product size of 238 bp
Rv, GAPDH		CGCTGATCACGACCTTCTTC	
OL14	pTB010 on SSU	ATCGCGACACGTTATGTGAG (Annealing upstream of integration region on SSU	Integration screening on SSU
OL2380	pRIB on SSU	CATTCCGTGCGAAAGCCGG	Integration screening on SSU
OL4613	LINF_310031200	A**TCTAGA**TTATAAATCCAGTGCGATCG	Knocked out cell line screening – CRISPR-Cas9 Amplification for re-expression
OL4614	LINF_310031200	TAT**AAGCTT**CTGTCATCACTCTTGTTAATGCG	Knocked out cell line screening – CRISPR-Cas9 Amplification for re-expression
OL4615	LINF_310031300	A**TCTAGA**CTAGAGGGCGACGTGCTCAT	Knocked out cell line screening – CRISPR-Cas9 Amplification for re-expression
OL4616	LINF_310031300	TAT**AAGCTT**ATGGCTCGAGCTCGTTTCC	Knocked out cell line screening – CRISPR-Cas9 Amplification for re-expression
OL4617	LINF_310031400	A**TCTAGA**CTGCTACGCGCTCCTGTG	Knocked out cell line screening – CRISPR-Cas9 Amplification for re-expression
OL4618	LINF_310031400	TAT**AAGCTT**ATGACCCTGCAGTGCGAT	Knocked out cell line screening – CRISPR-Cas9 Amplification for re-expression
OL4619	LINF_310031500	A**TCTAGA**CAGATTGCAGAATTCACGC	Knocked out cell line screening – CRISPR-Cas9 Amplification for re-expression
OL4620	LINF_310031500	TAT**AAGCTT**GCGTGGTTATATACGTGAGCG	Knocked out cell line screening – CRISPR-Cas9 Amplification for re-expression
OL4621	MSL deletion	AGTTGAGTCTGCTCCGGTG	Knocked out cell line screening – CRISPR-Cas9
OL4622	MSL deletion	TTCACGTCACGGCCAAAG	Knocked out cell line screening – CRISPR-Cas9
OL4747	pRIB	GCAGAAGCACTGTCGAATAGG	Integration screening on SSU
OL4867	hSpCas9	GCCTCCCTGGGCACATACCAC	hSpCas9 screening
OL4868	hSpCas9	CCAGCTGTCTCTTGATGAAGCCGG	hSpCas9 screening
OL8987	pTB010 on SSU	CGTCCGAGGGCAAAGGAATA (Annealing on construct HYG CDS)	Integration screening on SSU
OL9328	pPLOTv1 blast mNeonGreen-blast	AGAACAGGGGCATCTTGAGC	Repair template (tagged cell lines) integration screening – CRISPR-Cas9
OL9369	pTBlast_v1 and pTPuro_v1	GCAGCAGGTCTGCATTATAC	Repair templates (knocked out cell lines) integration screening – CRISPR-Cas9
OL9972	LINF_310031200	CTGGTCCTCCTCTGCACCGTCGCGCTTCCGgtataatgcagacctgctgc	Upstream forward primer – CRISPR-Cas9
OL9973	LINF_310031200	CTGGTCCTCCTCTGCACCGTCGCGCTTCCGTAATACGACTCACTATAAAACTGGAAG**GAGCTTCGACGG**gtataatgcagacctgctgc	Upstream forward primer with barcode – CRISPR-Cas9
OL9974	LINF_310031200	GTGGCGCTGCGCAATGAGGGCGACGGCCATactacccgatcctgatccag	Upstream reverse primer – CRISPR-Cas9
OL9975	LINF_310031200	gaaattaatacgactcactataggTTGCTCCATCATGCCGCAGAgttttagagctagaaatagc	5' sgRNA primer – CRISPR-Cas9
OL9976	LINF_310031200	GCCTCCAAACAAACAAAAGAAAAGTGGCTGccaatttgagagacctgtgc	Downstream reverse primer – CRISPR-Cas9

OL ID	Target	Oligonucleotide sequence (5’ » 3’)	Description
OL9977	LINF_310031200	gaaattaatacgactcactataggCGTGGTTGCTTTCAAGGACGgttttagagctagaaatagc	3' sgRNA primer – CRISPR-Cas9
OL9978	LINF_310031300	CCTTTCCTCTCCACACCGTGAAGCAAACCTgtataatgcagacctgctgc	Upstream forward primer – CRISPR-Cas9
OL9979	LINF_310031300	CCTTTCCTCTCCACACCGTGAAGCAAACCTTAATACGACTCACTATAAAACTGGAAG**CTATAATATTAA**gtataatgcagacctgctgc	Upstream forward primer with barcode – CRISPR-Cas9
OL9980	LINF_310031300	CAGAAGCTGAAGGAAACGAGCTCGAGCCATactacccgatcctgatccag	Upstream reverse primer – CRISPR-Cas9
OL9981	LINF_310031300	gaaattaatacgactcactataggTGTACAGCACGTAATGGCTGgttttagagctagaaatagc	5' sgRNA primer – CRISPR-Cas9
OL9982	LINF_310031300	GATAGGATCGAGAGGAGGCACAGGGGGAAAccaatttgagagacctgtgc	Downstream reverse primer – CRISPR-Cas9
OL9983	LINF_310031300	gaaattaatacgactcactataggAAGACGCTGCTTTCTTCTGCgttttagagctagaaatagc	3' sgRNA primer – CRISPR-Cas9
OL9984	LINF_310031400	CAAGCGCGAGGCTTATCGACTCACCAGCCCgtataatgcagacctgctgc	Upstream forward primer – CRISPR-Cas9
OL9985	LINF_310031400	CAAGCGCGAGGCTTATCGACTCACCAGCCCTAATACGACTCACTATAAAACTGGAAG**CTCGATCGACGG**gtataatgcagacctgctgc	Upstream forward primer with barcode – CRISPR-Cas9
OL9987	LINF_310031400	GCGGTAGAGCTCATCGCACTGCAGGGTCATactacccgatcctgatccag	Upstream reverse primer – CRISPR-Cas9
OL9988	LINF_310031400	gaaattaatacgactcactataggCTATGCAGCATACATGGGAAgttttagagctagaaatagc	5' sgRNA primer – CRISPR-Cas9
OL9989	LINF_310031400	GCGCGTCTATTCTAAATACAAGAAACACCAccaatttgagagacctgtgc	Downstream reverse primer – CRISPR-Cas9
OL9990	LINF_310031400	gaaattaatacgactcactataggGTTCCGTTGTGAATACTCGGgttttagagctagaaatagc	3' sgRNA primer – CRISPR-Cas9
OL9991	LINF_310031500	CGTCTCTTCGCCGCCGCGTCCGCCAGGCCTgtataatgcagacctgctgc	Upstream forward primer – CRISPR-Cas9
OL9992	LINF_310031500	CGTCTCTTCGCCGCCGCGTCCGCCAGGCCTTAATACGACTCACTATAAAACTGGAAG**TATATCGATGCC**gtataatgcagacctgctgc	Upstream forward primer with barcode – CRISPR-Cas9
OL9993	LINF_310031500	GGTGAGAGTGGACGATATGGCGCGGCGCATactacccgatcctgatccag	Upstream reverse primer – CRISPR-Cas9
OL9994	LINF_310031500	gaaattaatacgactcactataggATATGGTGGGTCTTCGTTGGgttttagagctagaaatagc	5' sgRNA primer – CRISPR-Cas9
OL9995	LINF_310031500	CGTGCGCACAAAGAACTCAGAGCCTCTCCTccaatttgagagacctgtgc	Downstream reverse primer – CRISPR-Cas9
OL9996	LINF_310031500	gaaattaatacgactcactataggACGTCTACATGCCCCTCATGgttttagagctagaaatagc	3' sgRNA primer – CRISPR-Cas9
OL10358	LINF_31MSL	ATATATCCATACATATCGATGTGTATATATgtataatgcagacctgctgc	Upstream forward primer – CRISPR-Cas9
OL10359	LINF_31MSL	ATATATCCATACATATCGATGTGTATATATTAATACGACTCACTATAAAACTGGAAG**ATACATACCATT**gtataatgcagacctgctgc	Upstream forward primer with barcode – CRISPR-Cas9
OL10360	LINF_31MSL	gaaattaatacgactcactataggGTAGGTGTGCTGAGGTGTCTgttttagagctagaaatagc	5' sgRNA primer – CRISPR-Cas9
OL10361	LINF_31MSL	CGATGCAGGTCTAGGGCAGTGGATTCACCAccaatttgagagacctgtgc	Downstream reverse primer – CRISPR-Cas9
OL10362	LINF_31MSL	gaaattaatacgactcactataggAACCGACGAGCAGACGGGAGgttttagagctagaaatagc	3' sgRNA primer – CRISPR-Cas9
OL10376	LINF_310031300	TCGTCACGTCGATAGTCAGC	Knocked out cell line screening – CRISPR-Cas9
OL10377	LINF_310031300	CGTAGCAGTTCCGTTGTGAA	Knocked out cell line screening – CRISPR-Cas9
OL10640	LINF_310031200	CAGCGATGCGAGCCGATCGCACTGGATTTAggttctggtagtggttccgg	Downstream forward primer – CRISPR-Cas9
OL10641	LINF_310031300	CGCTTTGGTTCCTATGAGCACGTCGCCCTCggttctggtagtggttccgg	Downstream forward primer – CRISPR-Cas9
OL10642	LINF_310031400	CTGCAACACGTGATGCCGCACAGGAGCGCGggttctggtagtggttccgg	Downstream forward primer – CRISPR-Cas9
OL10643	LINF_310031500	CTGGAACGCCTCAAGAGCCGCTCTCGTAAGggttctggtagtggttccgg	Downstream forward primer – CRISPR-Cas9
OL10855	LINF_310031200	GCGGAAGAGCCTCTACAAGA	Knocked out cell line screening – CRISPR-Cas9

Fw, forward oligonucleotide; Rv, reverse oligonucleotide. **AAGCTT**, restriction site for HindIII enzyme; **TCTAGA**, restriction site for XbaI enzyme. Barcodes are highlighted in bold/red.

Supplementary Table 2: Read depth coverage differences between MSL mutants/MG11A and *L. infantum* T7/Cas9. Read depth coverage (RDC) of each coding sequence (CDS) were estimated, normalized per chromosome RDC, and compared between mutants/MG11A and T7/Cas9. All CDS with difference [mutants/MG11A - T7/Cas9] higher than 0.5 RDC, which corresponds to approximately one copy per haploid genome, are reported. Table with all CDS normalized was deposited on Figshare, DOI: https://doi.org/10.6084/m9.figshare.21318240.

Chr.	CDS position		Gene ID	Product Description	RDC				Note
	Start	End			MA01A	T7/Cas9	Comparison	Difference	
**Δ*nuc1* vs. T7/Cas9**									
LinJ.10	437644	438124	LINF_100015600	FKBP-type peptidyl-prolyl cis-trans isomerase - putative	0.75	0.45	0.96	0.51	---
LinJ.19	697774	698152	LINF_190022700	ATG8/AUT7/APG8/PAZ2 - putative	0.93	0.77	1.27	0.50	---
LinJ.21	7847	8150	LINF_210005100	histone H4	0.87	0.50	1.11	0.61	Multiple paralogues •
LinJ.22	777241	778756	LINF_220023200	hypothetical protein	5.66	4.42	5.40	0.98	Multi copy gene •
LinJ.27	1172825	1174127	LINF_270033890	hypothetical protein - conserved	3.77	3.22	4.01	0.79	Multi copy gene •
LinJ.28	244493	245153	LINF_280011400	ER lumen retaining receptor-like protein	0.86	1.08	0.46	-0.62	---
LinJ.30	93045	93492	LINF_300008000	hypothetical protein - conserved	0.83	0.50	1.14	0.64	---
LinJ.30	1351032	1351701	LINF_300043200	ribosomal P protein AGP2beta-1 - putative	0.73	0.71	1.23	0.52	---
LinJ.31	1181281	1182328	LINF_310031200	NUC1	0.80	0.71	0.00	-0.71	MSL gene
LinJ.34	787427	788009	LINF_340023600	amastin-like surface protein - putative	1.27	0.69	1.38	0.69	Multi copy gene / Multiple paralogues •
LinJ.35	156279	159228	LINF_350010100	proteophosphoglycan ppg3 - putative	3.98	3.43	3.96	0.53	Multi copy gene •
LinJ.35	168215	175547	LINF_350010200	proteophosphoglycan ppg3 - putative	10.24	8.71	9.21	0.50	Multi copy gene •
**Δ*nuc2* vs. T7/Cas9**									
LinJ.12	455587	456073	LINF_120014800	hypothetical protein - conserved	0.56	0.43	0.95	0.52	Multiple paralogues
LinJ.27	1172825	1174127	LINF_270033890	hypothetical protein - conserved	3.77	3.22	3.74	0.52	Multi copy gene •
LinJ.31	1184204	1185341	LINF_310031300	NUC2	0.76	0.78	0.00	-0.78	MSL gene
LinJ.36	456312	456735	LINF_360017700	hypothetical protein - conserved	0.67	1.07	0.51	-0.56	---
**Δ*hlp* vs. T7/Cas9**									
LinJ.10	176091	176334	LINF_100009200	hypothetical protein	0.81	0.75	0.14	-0.61	Multiple paralogues
LinJ.10	231389	231845	LINF_100010500	hypothetical protein	2.11	1.76	2.31	0.54	Multi copy gene •
LinJ.22	777241	778756	LINF_220023200	hypothetical protein	5.66	4.42	5.16	0.74	Multi copy gene •
LinJ.25	387568	387880	LINF_250016300	hypothetical protein	0.70	0.35	0.93	0.58	•
LinJ.31	1185826	1188553	LINF_310031400	HLP	0.80	0.73	0.00	-0.73	MSL gene
LinJ.32	964910	965129	LINF_320031400	hypothetical protein	0.89	0.46	1.02	0.56	---
**Δ*tei* vs. T7/Cas9**									
LinJ.01	78067	78247	LINF_010007900	4F5 protein family - putative	0.73	0.41	0.93	0.51	---

Chr.	CDS position		Gene ID	Product Description	RDC				Note
	Start	End			MA01A	T7/Cas9	Comparison	Difference	
LinJ.08	492341	493862	LINF_080017100	Zinc finger - C3HC4 type (RING finger) - putative	1.51	1.34	1.89	0.55	Multi copy gene
LinJ.21	7847	8150	LINF_210005100	histone H4	0.87	0.50	1.06	0.56	Multiple paralogues •
LinJ.22	777241	778756	LINF_220023200	hypothetical protein	5.66	4.42	6.72	2.30	Multi copy gene •
LinJ.25	114783	115389	LINF_250009100	hypothetical protein - conserved	0.79	0.37	0.96	0.60	---
LinJ.27	1172825	1174127	LINF_270033890	hypothetical protein - conserved	3.77	3.22	4.76	1.54	Multi copy gene •
LinJ.31	1191356	1192406	LINF_310031500	TEI	0.81	0.70	0.08	-0.62	MSL gene
LinJ.32	1462495	1462894	LINF_320044900	kinetoplast-associated protein p18-2 - putative	0.95	0.56	1.06	0.51	---
LinJ.33	1520147	1520558	LINF_330042600	h1 histone-like protein	0.84	0.55	1.12	0.58	---
LinJ.34	333051	333534	LINF_340012700	Predicted membrane protein - putative	0.83	0.37	0.93	0.57	---
LinJ.35	1283	3371	LINF_350005000	phosphoglycan beta 1,3 galactosyltransferase 7	0.74	0.56	1.16	0.60	Multi copy gene
LinJ.35	156279	159228	LINF_350010100	proteophosphoglycan ppg3 - putative	3.98	3.43	4.04	0.61	Multi copy gene •
LinJ.35	168215	175547	LINF_350010200	proteophosphoglycan ppg3 - putative	10.24	8.71	9.42	0.71	Multi copy gene •
LinJ.35	1921447	1921678	LINF_350056500	Ribosomal protein L37e - putative	0.71	0.45	1.00	0.55	Multiple paralogues
LinJ.36	99013	99064	LINF_360008450	hypothetical protein	0.76	0.53	1.07	0.54	---
LinJ.36	2739457	2740183	LINF_360082400	PSP1 C-terminal conserved region containing protein - putative	0.84	0.51	1.03	0.52	Multiple paralogues
**Δ*nuc1/nuc2* vs. T7/Cas9**									
LinJ.21	572748	572988	LINF_210022300	hypothetical protein - conserved	0.75	0.44	1.08	0.64	Multiple paralogues
LinJ.31	1181281	1182328	LINF_310031200	NUC1	0.80	0.71	0.00	-0.71	MSL gene
LinJ.31	1184204	1185341	LINF_310031300	NUC2	0.76	0.78	0.00	-0.78	MSL gene
LinJ.34	787427	788009	LINF_340023600	amastin-like surface protein - putative	1.27	0.69	1.26	0.57	Multi copy gene / Multiple paralogues •
LinJ.35	168215	175547	LINF_350010200	proteophosphoglycan ppg3 - putative	10.24	8.71	9.42	0.71	Multi copy gene •
**Δ*msl* vs. T7/Cas9**									
LinJ.22	777241	778756	LINF_220023200	hypothetical protein	5.66	4.42	5.42	0.99	Multi copy gene •
LinJ.25	387568	387880	LINF_250016300	hypothetical protein	0.70	0.35	0.89	0.53	•
LinJ.27	1172825	1174127	LINF_270033890	hypothetical protein - conserved	3.77	3.22	3.87	0.66	Multi copy gene •
LinJ.31	1181281	1182328	LINF_310031200	NUC1	0.80	0.71	0.00	-0.71	MSL gene
LinJ.31	1184204	1185341	LINF_310031300	NUC2	0.76	0.78	0.00	-0.78	MSL gene
LinJ.31	1185826	1188553	LINF_310031400	HLP	0.80	0.73	0.00	-0.73	MSL gene
LinJ.31	1191356	1192406	LINF_310031500	TEI	0.81	0.70	0.08	-0.62	MSL gene
LinJ.35	156279	159228	LINF_350010100	proteophosphoglycan ppg3 - putative	3.98	3.43	3.97	0.54	Multi copy gene •
LinJ.35	168215	175547	LINF_350010200	proteophosphoglycan ppg3 - putative	10.24	8.71	9.35	0.64	Multi copy gene •

Chr.	CDS position		Gene ID	Product Description	RDC				Note
	Start	End			MA01A	T7/Cas9	Comparison	Difference	
**MG11A vs. T7/Cas9**									
LinJ.02	7750	8053	LINF_020005100	histone H4	1.38	1.57	0.97	-0.60	DNA binding; Protein heterodimerization activity
LinJ.02	90962	92927	LINF_020006900	phosphoglycan beta 1 -3 galactosyltransferase	1.67	1.35	2.20	0.84	Protein glycosylation
LinJ.02	99642	101961	LINF_020007000	phosphoglycan beta 1 -3 galactosyltransferase	2.01	1.76	2.64	0.88	Protein glycosylation
LinJ.08	289982	290597	LINF_080011900	amastin-like protein	0.75	0.49	1.48	0.99	---
LinJ.08	295606	296212	LINF_080012000	amastin-like protein	0.94	0.67	2.49	1.82	---
LinJ.08	300237	300840	LINF_080012100	Amastin surface glycoprotein - putative	0.48	0.38	1.06	0.68	---
LinJ.08	304862	305465	LINF_080012200	Amastin surface glycoprotein - putative	0.52	0.36	1.00	0.64	---
LinJ.08	309446	310049	LINF_080012300	Amastin surface glycoprotein - putative	0.46	0.40	1.04	0.64	---
LinJ.08	313959	314571	LINF_080012400	amastin-like protein	0.48	0.38	0.98	0.59	---
LinJ.08	337858	338386	LINF_080012900	Amastin-like protein	1.53	1.15	1.67	0.52	---
LinJ.10	231389	231845	LINF_100010500	hypothetical protein	2.11	1.76	2.27	0.51	Cell adhesion; Proteolysis
LinJ.10	242240	244040	LINF_100010900	GP63 - leishmanolysin	2.25	1.86	2.38	0.52	Cell adhesion; Proteolysis
LinJ.10	245287	247087	LINF_100011000	GP63 - leishmanolysin	2.12	1.79	2.33	0.53	Cell adhesion; Proteolysis
LinJ.10	255010	256810	LINF_100011300	GP63 - leishmanolysin	2.08	1.75	2.25	0.50	Cell adhesion; Proteolysis
LinJ.14	266070	266970	LINF_140012400	fatty acid elongase - putative	0.85	0.57	1.71	1.14	Fatty acid elongase activity
LinJ.15	522568	524044	LINF_150020000	nucleoside transporter 1 - putative	0.37	0.30	0.84	0.55	Nucleoside transmembrane transport
LinJ.15	526293	527769	LINF_150020100	nucleoside transporter 1 - putative	0.31	0.22	0.88	0.66	Nucleoside transmembrane transport
LinJ.19	361121	361487	LINF_190013900	ATG8/AUT7/APG8/PAZ2 - putative	0.32	0.24	0.74	0.50	---
LinJ.19	364703	365069	LINF_190014100	ATG8/AUT7/APG8/PAZ2 - putative	0.34	0.36	0.86	0.50	---
LinJ.22	777241	778756	LINF_220023200	hypothetical protein	5.66	4.42	5.76	1.34	---
LinJ.23	572031	572403	LINF_230020400	hypothetical protein - conserved	0.78	0.44	0.99	0.55	---
LinJ.24	211299	211797	LINF_240010800	hypothetical protein	0.79	0.46	0.96	0.51	---
LinJ.25	387568	387880	LINF_250016300	hypothetical protein	0.70	0.35	0.90	0.55	---
LinJ.25	886708	887011	LINF_250031800	histone H4	0.28	0.24	0.90	0.66	DNA binding;protein heterodimerization activity
LinJ.27	1172825	1174127	LINF_270033890	hypothetical protein - conserved	3.77	3.22	3.79	0.57	---
LinJ.28	351787	352168	LINF_280015100	Tctex-1 family - putative	0.89	0.44	0.94	0.51	---
LinJ.28	841412	842462	LINF_280027700	glycerophosphoryl diester phosphodiesterase - putative	0.80	0.41	0.91	0.50	Lipid metabolic process
LinJ.30	220944	221145	LINF_300011800	40S ribosomal protein S30 - putative	0.83	0.67	1.21	0.54	Translation
LinJ.31	1017453	1017756	LINF_310027800	hypothetical protein	0.72	0.44	0.96	0.52	---
LinJ.31	1181281	1182328	LINF_310031200	NUC1	0.80	0.71	0.00	-0.71	MSL gene: DNA catabolic process
LinJ.31	1184204	1185341	LINF_310031300	NUC2	0.76	0.78	0.00	-0.78	MSL gene: DNA catabolic process
LinJ.31	1185826	1188553	LINF_310031400	HLP	0.80	0.73	0.00	-0.73	MSL gene: ATP binding

Chr.	CDS position		Gene ID	Product Description	RDC				Note
	Start	End			MA01A	T7/Cas9	Comparison	Difference	
LinJ.31	1191356	1192406	LINF_310031500	TEI	0.81	0.70	0.10	-0.60	MSL gene: catalytic activity
LinJ.32	964910	965129	LINF_320031400	hypothetical protein	0.89	0.46	1.01	0.55	---
LinJ.33	327305	328637	LINF_330015200	beta tubulin	1.61	1.17	1.69	0.52	Microtubule-based process
LinJ.34	333051	333534	LINF_340012700	Predicted membrane protein - putative	0.83	0.37	0.87	0.50	---
LinJ.34	745976	746594	LINF_340023000	Amastin surface glycoprotein - putative	1.07	0.69	1.22	0.53	---
LinJ.34	749230	749848	LINF_340023100	Amastin surface glycoprotein - putative	0.94	0.73	1.34	0.61	---
LinJ.34	770988	771570	LINF_340023400	amastin-like surface protein - putative	1.22	0.92	1.44	0.53	---
LinJ.34	787427	788009	LINF_340023600	amastin-like surface protein - putative	1.27	0.69	1.38	0.69	---
LinJ.34	1258241	1258829	LINF_340034600	Amastin surface glycoprotein - putative	1.71	1.52	2.05	0.54	---
LinJ.35	156279	159228	LINF_350010100	proteophosphoglycan ppg3 - putative	3.98	3.43	4.79	1.35	Immune response; Toll-like receptor signaling pathway
LinJ.35	168215	175547	LINF_350010200	proteophosphoglycan ppg3 - putative	10.24	8.71	11.12	2.41	Immune response; Toll-like receptor signaling pathway
LinJ.36	2735520	2736015	LINF_360082100	U1A small nuclear ribonucleoprotein - putative	0.94	0.38	0.89	0.52	Nucleic acid binding

Chr., chromosome. Note: •, CDS that showed RDC difference > 0.5 in more than one MSL knockout line; MSL, miltefosine sensitive locus.

Supplementary Table 3: Summary of variants identified in coding sequence region of the *L. infantum* knockout mutants against T7/Cas9. Variants were filtered for minor allele count >3 to monomorphic variants. Table with all variants identified was deposited on Figshare, DOI: https://doi.org/10.6084/m9.figshare.21334602.

Chr.	Position	Reference sequence (0)	Altered sequence (1;2;3)	Quality score	Variant impact	Genomic feature	Associated gene	Genotype		
								MA01A	T7/Cas9	Δ*nuc1*
LinJ.10	175781	T	G	319.799	Moderate	CDS	LINF_100009100	0/1	0/1	0/0
LinJ.11	20258	G	A	327.929	Moderate	CDS	LINF_110005600	0/1	1/1	0/0
LinJ.11	508343	G	A	165.248	Moderate	CDS	LINF_110018350	0/1	0/1	0/0
LinJ.11	508355	CCGGA	ACGGG	864.956	Moderate	CDS	LINF_110018350	0/1	0/1	0/0
LinJ.12	407488	A	G	635.555	Moderate	CDS	LINF_120013300	0/1	0/1	0/0
LinJ.12	407493	G	A	559.85	Moderate	CDS	LINF_120013300	0/1	0/1	0/0
LinJ.14	503382	C	A	685.08	Moderate	CDS	LINF_140017400	0/1	0/1	0/0
LinJ.14	511767	AAC	AAG	353.211	Moderate	CDS	LINF_140017500	0/1	0/1	0/0
LinJ.14	513700	A	G	125.698	Moderate	CDS	LINF_140017500	0/0	0/0	0/1
LinJ.14	517675	C	G	368.782	Moderate	CDS	LINF_140017500	0/1	0/1	0/0
LinJ.16	594184	G	T	206.471	Moderate	CDS	LINF_160020700	1/1	0/0	0/1
LinJ.22	395729	GGCTGTTGGCCCGCTCTCCGT	GGCTGTTGGCCCGCTCTCCGTTGGCCCG CAGTCTGT	814.536	Moderate	CDS	LINF_220014400	0/1	0/0	1/1
LinJ.22	603126	GAG	CAC	121.468	Moderate	CDS	LINF_220019300	0/1	0/1	0/0
LinJ.22	604346	T	C	143.946	Moderate	CDS	LINF_220019300	0/0	0/1	1/1
LinJ.23	10055	A	C	40.3924	Moderate	CDS	LINF_230005300	0/0	0/0	0/1
LinJ.31	1151875	G	C	38.116	Moderate	CDS	LINF_310030400	0/1	0/1	0/0
LinJ.32	892355	AAGGC	AAGGT	76.1411	Moderate	CDS	LINF_320029100	0/0	0/0	0/1
LinJ.35	158648	T	C	508.892	Moderate	CDS	LINF_350010100	0/1	0/1	1/1
LinJ.35	158716	G	C	31.1805	Moderate	CDS	LINF_350010100	0/0	0/0	0/1
LinJ.35	171534	TCGC	GCGT	50.1271	Moderate	CDS	LINF_350010200	0/1	0/1	0/0
LinJ.36	2068365	C	G	149.977	Moderate	CDS	LINF_360062000	1/1	0/1	0/0
LinJ.02	12368	G	A	66.2392	Low	CDS	LINF_020005300	0/0	0/0	0/1
LinJ.14	510767	C	T	64.7176	Low	CDS	LINF_140017500	0/1	0/1	1/1
LinJ.14	515174	G	A	39.4405	Low	CDS	LINF_140017500	0/1	0/1	0/0
LinJ.14	515198	A	G	468.366	Low	CDS	LINF_140017500	0/1	0/1	0/0
LinJ.15	163452	G	A	52.7764	Low	CDS	LINF_150009800	0/1	0/1	0/0
LinJ.27	1173671	T	G	1685.61	Low	CDS	LINF_270033890	0/1	0/1	0/0
LinJ.32	892325	GAGC	AAGG	50.7191	Low	CDS	LINF_320029100	0/1	0/1	0/0
LinJ.35	173975	G	A	406.402	Low	CDS	LINF_350010200	0/1	0/1	0/0
LinJ.35	174494	G	A	110.047	Low	CDS	LINF_350010200	0/1	0/1	0/0

Chr.	Position	Reference sequence (0)	Altered sequence (1;2;3)	Quality score	Variant impact	Genomic feature	Associated gene	Genotype		
								MA01A	T7/Cas9	Δ*nuc2*
LinJ.02	346989	C	T	392.723	Moderate	CDS	LINF_020012400	1/1	1/1	0/1
LinJ.11	508330	G	C	42.6178	Moderate	CDS	LINF_110018350	0/0	0/0	0/1
LinJ.11	508337	A	G	46.3174	Moderate	CDS	LINF_110018350	0/0	0/0	0/1
LinJ.13	293651	T	C	103.113	Moderate	CDS	LINF_130013400	0/1	0/1	0/0
LinJ.14	513700	A	G	125.698	Moderate	CDS	LINF_140017500	0/0	0/0	0/1
LinJ.14	517590	A	G	271.366	Moderate	CDS	LINF_140017500	0/1	0/1	0/0
LinJ.15	164728	A	G	82.3111	Moderate	CDS	LINF_150009800	0/1	0/1	0/0
LinJ.23	10055	A	C	40.3924	Moderate	CDS	LINF_230005300	0/0	0/0	0/1
LinJ.31	1151875	G	C	38.116	Moderate	CDS	LINF_310030400	0/1	0/1	0/0
LinJ.32	892355	AAGGC	AAGGT	76.1411	Moderate	CDS	LINF_320029100	0/0	0/0	0/1
LinJ.33	1445871	G	T	615.552	Moderate	CDS	LINF_330040700	0/1	0/1	0/0
LinJ.34	319484	C	G	474.818	Moderate	CDS	LINF_340012400	0/1	0/1	0/0
LinJ.35	158493	GTCGG	ATCGC	904.01	Moderate	CDS	LINF_350010100	0/1	0/1	0/0
LinJ.35	158882	A	G	1900.74	Moderate	CDS	LINF_350010100	0/1	0/1	0/0
LinJ.14	510767	C	T	64.7176	Low	CDS	LINF_140017500	0/1	0/1	0/0
LinJ.14	517676	GA	GC	253.8	Low	CDS	LINF_140017500	0/1	0/1	0/0
LinJ.15	163452	G	A	52.7764	Low	CDS	LINF_150009800	0/1	0/1	0/0
LinJ.22	599299	T	G	109.915	Low	CDS	LINF_220019200	0/1	0/1	0/0
LinJ.32	892325	GAGC	AAGG	50.7191	Low	CDS	LINF_320029100	0/1	0/1	0/0
LinJ.35	158427	G	A	429.114	Low	CDS	LINF_350010100	0/1	0/1	0/0
LinJ.35	171638	C	G	47.6726	Low	CDS	LINF_350010200	0/0	0/0	0/1

Chr.	Position	Reference sequence (0)	Altered sequence (1;2;3)	Quality score	Variant impact	Genomic feature	Associated gene	Genotype		
								MA01A	T7/Cas9	Δ*hlp*
LinJ.11	20258	G	A	327.929	Moderate	CDS	LINF_110005600	0/1	1/1	0/0
LinJ.11	508337	A	G	46.3174	Moderate	CDS	LINF_110018350	0/0	0/0	0/1
LinJ.13	293651	T	C	103.113	Moderate	CDS	LINF_130013400	0/1	0/1	0/0
LinJ.14	503382	C	A	685.08	Moderate	CDS	LINF_140017400	0/1	0/1	0/0
LinJ.14	513700	A	G	125.698	Moderate	CDS	LINF_140017500	0/0	0/0	0/1
LinJ.15	164644	GCGG	ACGC	180.618	Moderate	CDS	LINF_150009800	0/1	0/1	1/1
LinJ.16	594184	G	T	206.471	Moderate	CDS	LINF_160020700	1/1	0/0	0/1
LinJ.22	604346	T	C	143.946	Moderate	CDS	LINF_220019300	0/0	0/1	1/1
LinJ.23	10055	A	C	40.3924	Moderate	CDS	LINF_230005300	0/0	0/0	0/1
LinJ.31	1151875	G	C	38.116	Moderate	CDS	LINF_310030400	0/1	0/1	0/0

Chr.	Position	Reference sequence (0)	Altered sequence (1;2;3)	Quality score	Variant impact	Genomic feature	Associated gene	Genotype		
								MA01A	T7/Cas9	Δ*hlp*
LinJ.32	892355	AAGGC	AAGGT	76.1411	Moderate	CDS	LINF_320029100	0/0	0/0	0/1
LinJ.35	158992	GTCGGCGTCCTCGTCGTCT	GTCGGCGTCGTCGTCGTCT	86.1395	Moderate	CDS	LINF_350010100	0/1	0/1	0/0
LinJ.35	171534	TCGC	GCGT	50.1271	Moderate	CDS	LINF_350010200	0/1	0/1	1/1
LinJ.02	12368	G	A	66.2392	Low	CDS	LINF_020005300	0/0	0/0	0/1
LinJ.14	510767	C	T	64.7176	Low	CDS	LINF_140017500	0/1	0/1	1/1
LinJ.35	171638	C	G	47.6726	Low	CDS	LINF_350010200	0/0	0/0	1/1

Chr.	Position	Reference sequence (0)	Altered sequence (1;2;3)	Quality score	Variant impact	Genomic feature	Associated gene	Genotype		
								MA01A	T7/Cas9	Δ*tei*
LinJ.11	20258	G	A	327.929	Moderate	CDS	LINF_110005600	0/1	1/1	0/0
LinJ.11	508889	ATATTC	ATGTTT	1825.67	Moderate	CDS	LINF_110018350	0/1	0/1	0/0
LinJ.14	297402	C	A	1501.65	Moderate	CDS	LINF_140013100	0/1	0/1	0/0
LinJ.14	513700	A	G	125.698	Moderate	CDS	LINF_140017500	0/0	0/0	0/1
LinJ.14	516523	G	C	109.984	Moderate	CDS	LINF_140017500	0/1	0/1	0/0
LinJ.15	164728	A	G	82.3111	Moderate	CDS	LINF_150009800	0/1	0/1	0/0
LinJ.22	396481	TCT	AGG	755.038	Moderate	CDS	LINF_220014400	0/1	0/1	1/1
LinJ.22	604346	T	C	143.946	Moderate	CDS	LINF_220019300	0/0	0/1	1/1
LinJ.23	10055	A	C	40.3924	Moderate	CDS	LINF_230005300	0/0	0/0	0/1
LinJ.31	1151875	G	C	38.116	Moderate	CDS	LINF_310030400	0/1	0/1	0/0
LinJ.31	1155400	C	T	2764.24	Moderate	CDS	LINF_310030400	0/1	0/1	0/0
LinJ.32	892355	AAGGC	AAGGT	76.1411	Moderate	CDS	LINF_320029100	0/0	0/0	0/1
LinJ.35	158716	G	C	31.1805	Moderate	CDS	LINF_350010100	0/0	0/0	1/1
LinJ.35	158992	GTCGGCGTCCTCGTCGTCT	GTCGGCGTCGTCGTCGTCT	86.1395	Moderate	CDS	LINF_350010100	0/1	0/1	0/0
LinJ.14	510767	C	T	64.7176	Low	CDS	LINF_140017500	0/1	0/1	1/1
LinJ.15	163452	G	A	52.7764	Low	CDS	LINF_150009800	0/1	0/1	0/0
LinJ.15	164532	C	G	1848.09	Low	CDS	LINF_150009800	0/1	0/1	1/1
LinJ.16	594218	A	G	2360.21	Low	CDS	LINF_160020700	0/1	0/1	1/1
LinJ.22	599299	T	G	109.915	Low	CDS	LINF_220019200	0/1	0/1	0/0
LinJ.32	892325	GAGC	AAGG	50.7191	Low	CDS	LINF_320029100	0/1	0/1	0/0
LinJ.35	171638	C	G	47.6726	Low	CDS	LINF_350010200	0/0	0/0	1/1
LinJ.35	173975	G	A	406.402	Low	CDS	LINF_350010200	0/1	0/1	0/0

Chr.	Position	Reference sequence (0)	Altered sequence (1;2;3)	Quality score	Variant impact	Genomic feature	Associated gene	Genotype		
								MA01A	T7/Cas9	Δ*nuc1/nuc2*
LinJ.11	508330	G	C	42.6178	Moderate	CDS	LINF_110018350	0/0	0/0	0/1
LinJ.11	508337	A	G	46.3174	Moderate	CDS	LINF_110018350	0/0	0/0	0/1
LinJ.13	293651	T	C	103.113	Moderate	CDS	LINF_130013400	0/1	0/1	0/0
LinJ.14	515275	C	G	2926.33	Moderate	CDS	LINF_140017500	1/1	1/1	0/1
LinJ.14	517339	C	G	63.399	Moderate	CDS	LINF_140017500	0/0	1/1	0/1
LinJ.15	164644	GCGG	ACGC	180.618	Moderate	CDS	LINF_150009800	0/1	0/1	0/0
LinJ.22	603126	GAG	CAC	121.468	Moderate	CDS	LINF_220019300	0/1	0/1	1/1
LinJ.22	778316	CGC	CGG	6045.78	Moderate	CDS	LINF_220023200	0/1	0/1	1/1
LinJ.23	10055	A	C	40.3924	Moderate	CDS	LINF_230005300	0/0	0/0	0/1
LinJ.32	892355	AAGGC	AAGGT	76.1411	Moderate	CDS	LINF_320029100	0/0	0/0	0/1
LinJ.35	158648	T	C	508.892	Moderate	CDS	LINF_350010100	0/1	0/1	1/1
LinJ.35	158716	G	C	31.1805	Moderate	CDS	LINF_350010100	0/0	0/0	1/1
LinJ.35	158992	GTCGGCGTCCTCGTCGTCT	GTCGGCGTCGTCGTCGTCT	86.1395	Moderate	CDS	LINF_350010100	0/1	0/1	0/0
LinJ.35	170238	GT	GC	266.015	Moderate	CDS	LINF_350010200	0/1	0/1	0/0
LinJ.14	510767	C	T	64.7176	Low	CDS	LINF_140017500	0/1	0/1	1/1
LinJ.14	515174	G	A	39.4405	Low	CDS	LINF_140017500	0/1	0/1	0/0
LinJ.32	892325	GAGC	AAGG	50.7191	Low	CDS	LINF_320029100	0/1	0/1	0/0
LinJ.35	173189	TTCAGCGTCGTCGTCGTC	GTCAGCGTCGTCGTCGTC	501.855	Low	CDS	LINF_350010200	1/1	0/1	0/0

Chr.	Position	Reference sequence (0)	Altered sequence (1;2;3)	Quality score	Variant impact	Genomic feature	Associated gene	Genotype		
								MA01A	T7/Cas9	Δ*msl*
LinJ.10	175781	T	G	319.799	Moderate	CDS	LINF_100009100	0/1	0/1	0/0
LinJ.11	20258	G	A	327.929	Moderate	CDS	LINF_110005600	0/1	1/1	0/0
LinJ.11	508330	G	C	42.6178	Moderate	CDS	LINF_110018350	0/0	0/0	0/1
LinJ.11	508337	A	G	46.3174	Moderate	CDS	LINF_110018350	0/0	0/0	0/1
LinJ.11	508889	ATATTC	ATGTTT	1825.67	Moderate	CDS	LINF_110018350	0/1	0/1	0/0
LinJ.14	297402	C	A	1501.65	Moderate	CDS	LINF_140013100	0/1	0/1	0/0
LinJ.14	513700	A	G	125.698	Moderate	CDS	LINF_140017500	0/0	0/0	0/1
LinJ.14	515275	C	G	2926.33	Moderate	CDS	LINF_140017500	1/1	1/1	0/1
LinJ.14	517339	C	G	63.399	Moderate	CDS	LINF_140017500	0/0	1/1	0/1
LinJ.16	594184	G	T	206.471	Moderate	CDS	LINF_160020700	1/1	0/0	0/1
LinJ.22	396481	TCT	AGG	755.038	Moderate	CDS	LINF_220014400	0/1	0/1	1/1
LinJ.22	604346	T	C	143.946	Moderate	CDS	LINF_220019300	0/0	0/1	1/1
LinJ.23	10055	A	C	40.3924	Moderate	CDS	LINF_230005300	0/0	0/0	0/1

Chr.	Position	Reference sequence (0)	Altered sequence (1;2;3)	Quality score	Variant impact	Genomic feature	Associated gene	Genotype		
								MA01A	T7/Cas9	Δ*msl*
LinJ.31	1151875	G	C	38.116	Moderate	CDS	LINF_310030400	0/1	0/1	0/0
LinJ.32	892355	AAGGC	AAGGT	76.1411	Moderate	CDS	LINF_320029100	0/0	0/0	0/1
LinJ.35	158648	T	C	508.892	Moderate	CDS	LINF_350010100	0/1	0/1	1/1
LinJ.35	158716	G	C	31.1805	Moderate	CDS	LINF_350010100	0/0	0/0	1/1
LinJ.35	158992	GTCGGCGTCCTCGTCGTCT	GTCGGCGTCGTCGTCGTCT	86.1395	Moderate	CDS	LINF_350010100	0/1	0/1	0/0
LinJ.35	170320	T	C	796.457	Moderate	CDS	LINF_350010200	0/1	0/1	0/0
LinJ.35	171957	T	C	463.911	Moderate	CDS	LINF_350010200	0/1	0/1	1/1
LinJ.02	12368	G	A	66.2392	Low	CDS	LINF_020005300	0/0	0/0	0/1
LinJ.14	515174	G	A	39.4405	Low	CDS	LINF_140017500	0/1	0/1	0/0
LinJ.14	516923	G	A	1349.11	Low	CDS	LINF_140017500	0/1	0/1	1/1
LinJ.27	1173671	T	G	1685.61	Low	CDS	LINF_270033890	0/1	0/1	0/0
LinJ.32	892325	GAGC	AAGG	50.7191	Low	CDS	LINF_320029100	0/1	0/1	0/0
LinJ.35	171638	C	G	47.6726	Low	CDS	LINF_350010200	0/0	0/0	1/1

Chr., chromosome. CDS, coding sequence.

Supplementary Table 4: Summary of variants identified in coding sequence region of the *L. infantum* MG11A against T7/Cas9. Variants were filtered for minor allele count >3 to monomorphic variants. Table with all variants identified was deposited on Figshare, DOI: https://doi.org/10.6084/m9.figshare.21334602.

Chr.	Position	Reference sequence (0)	Altered sequence (1;2;3)	Quality score	Variant impact	Genomic feature	Associated gene	Genotype		
								MA01A	T7/Cas9	MG11A
LinJ.02	346989	C	T	392.723	Moderate	CDS	LINF_020012400	1/1	1/1	0/0
LinJ.07	48196	G	A	5073.36	Moderate	CDS	LINF_070006200	0/1	0/1	0/0
LinJ.11	508337	A	G	46.3174	Moderate	CDS	LINF_110018350	0/0	0/0	0/1
LinJ.11	508889	ATATTC	ATGTTT	1825.67	Moderate	CDS	LINF_110018350	0/1	0/1	0/0
LinJ.14	297402	C	A	1501.65	Moderate	CDS	LINF_140013100	0/1	0/1	0/0
LinJ.14	511767	AAC	AAG	353.211	Moderate	CDS	LINF_140017500	0/1	0/1	0/0
LinJ.14	515275	C	G	2926.33	Moderate	CDS	LINF_140017500	1/1	1/1	0/1
LinJ.14	517590	A	G	271.366	Moderate	CDS	LINF_140017500	0/1	0/1	0/0
LinJ.22	396481	TCT	AGG	755.038	Moderate	CDS	LINF_220014400	0/1	0/1	1/1
LinJ.26	848805	A	G	554.72	Moderate	CDS	LINF_260027000	0/1	0/1	0/0
LinJ.28	400404	C	G	2857.97	Moderate	CDS	LINF_280016400	0/1	0/1	0/0
LinJ.31	41621	C	T	2443.56	Moderate	CDS	LINF_310006600	0/1	0/1	0/0
LinJ.31	1151875	G	C	38.116	Moderate	CDS	LINF_310030400	0/1	0/1	0/0
LinJ.31	1155400	C	T	2764.24	Moderate	CDS	LINF_310030400	0/1	0/1	0/0
LinJ.32	414996	A	G	4397.35	Moderate	CDS	LINF_320016100	0/1	0/1	0/0
LinJ.33	1445871	G	T	615.552	Moderate	CDS	LINF_330040700	0/1	0/1	0/0
LinJ.34	319484	C	G	474.818	Moderate	CDS	LINF_340012400	0/1	0/1	0/0
LinJ.35	158493	GTCGG	ATCGC	904.01	Moderate	CDS	LINF_350010100	0/1	0/1	0/0
LinJ.35	171534	TCGC	GCGT	50.1271	Moderate	CDS	LINF_350010200	0/1	0/1	0/0
LinJ.35	173776	C	T	5759.3	Moderate	CDS	LINF_350010200	0/1	0/1	0/0
LinJ.35	173866	C	T	1301.79	Moderate	CDS	LINF_350010200	0/1	0/1	0/0
LinJ.11	512015	T	C	2050.01	Low	CDS	LINF_110018350	0/1	0/1	0/0
LinJ.14	515174	G	A	39.4405	Low	CDS	LINF_140017500	0/1	0/1	0/0
LinJ.15	163452	G	A	52.7764	Low	CDS	LINF_150009800	0/1	0/1	0/0
LinJ.31	112149	C	T	10083.9	Low	CDS	LINF_310008900	0/1	0/1	0/0
LinJ.32	892325	GAGC	AAGG	50.7191	Low	CDS	LINF_320029100	0/1	0/1	0/0
LinJ.35	158427	G	A	429.114	Low	CDS	LINF_350010100	0/1	0/1	0/0
LinJ.35	171638	C	G	47.6726	Low	CDS	LINF_350010200	0/0	0/0	1/1
LinJ.35	172457	G	A	17436.4	Low	CDS	LINF_350010200	0/1	0/1	0/0
LinJ.35	173975	G	A	406.402	Low	CDS	LINF_350010200	0/1	0/1	0/0

Chr., chromosome. CDS, coding sequence.

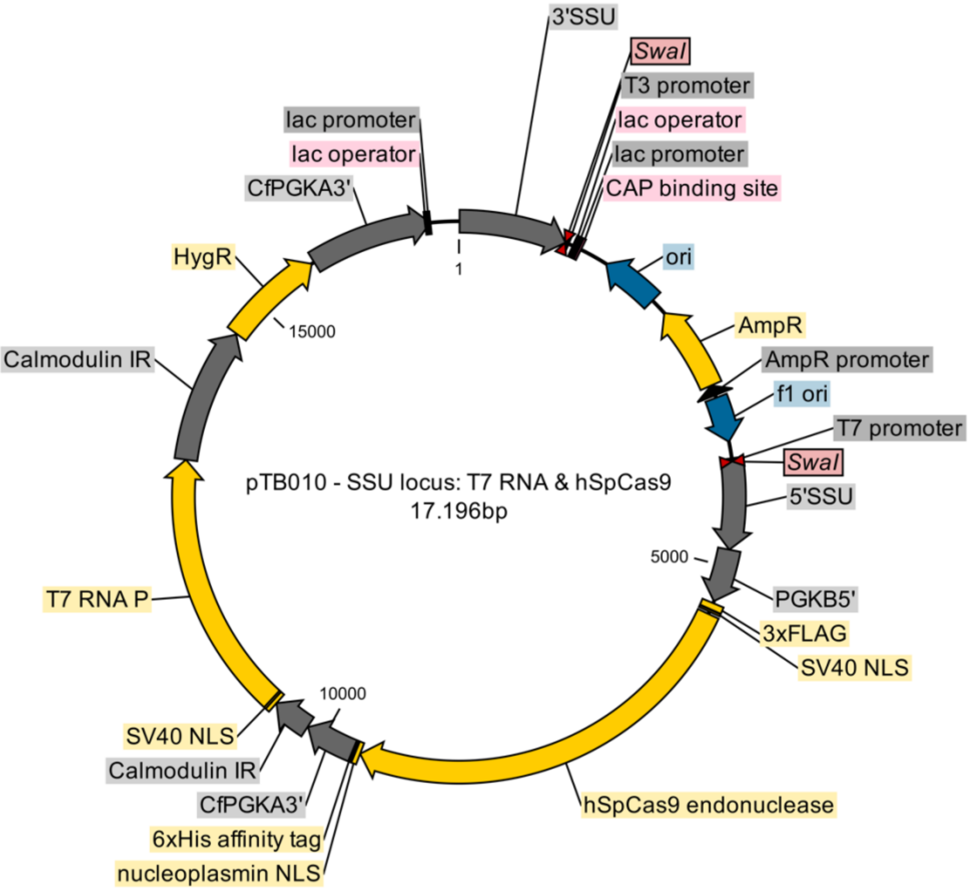

Supplementary Figure 1: Map of the plasmid pTB010. Plasmid used to generate the *L. infantum* cell line expressing T7 RNA polymerase and Cas9 endonuclease. SSU, homologous arm to integration on ribosomal locus of *Leishmania* spp.; T3 promoter, promoter for bacteriophage T3 RNA polymerase; lac operator, the lac repressor binds to the lac operator to inhibit transcription in *E. coli* (this inhibition can be relieved by adding lactose or isopropyl-beta-D-thiogalactopyranoside (IPTG)); lac promoter, promoter for the *E. coli* lac operon; CAP binding site, *E. coli* catabolite activator protein (CAP binding activates transcription in the presence of cAMP); ori, high-copy-number ColE1/pMB1/pBR322/pUC origin of replication; AmpR, product beta-lactamase to confer resistance to ampicillin, carbenicillin, and related antibiotics; f1 ori, f1 bacteriophage origin of replication (arrow indicates direction of (+) strand synthesis); T7 promoter, promoter for bacteriophage T7 RNA polymerase; 3xFLAG, three tandem FLAG(R) epitope tags, followed by an enterokinase cleavage site; NLS40, nuclear localization signal of SV40 large T antigen; hSpCas9 endonuclease, Cas9 (Csn1) endonuclease from the Streptococcus pyogenes Type II CRISPR/Cas system; nucleoplasmin NLS, bipartite nuclear localization signal from nucleoplasmin; T7 RNA P, T7 RNA polymerase; HygR, aminoglycoside phosphotransferase from *E. coli* (gene aph(4)-Ia to confers resistance to hygromycin); SwaI, restriction site for SwaI enzyme. The plasmid map was designed on CLC Main workbench v.20.0.3.

(a)

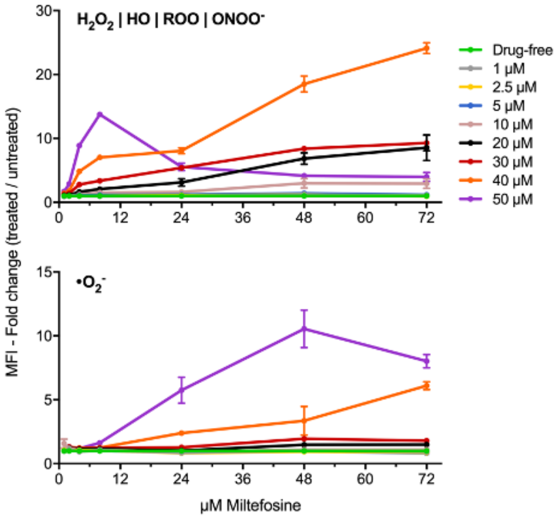

(b)

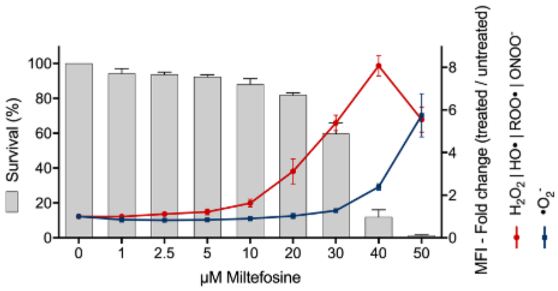

Supplementary Figure 2: Concentration and time effect of miltefosine in ROS accumulation and cell viability on promastigote stage of *L. infantum* parasite. The Brazilian reference strain of *L. infantum* MHOM/BR/74/PP75 was treated with different miltefosine concentrations over 24 h. (a) The reactive oxygen species were measured using the probes H_2DCFDA (H_2O_2, HO•, ROO• and $ONOO^-$) and MitoSOXTM (•O_2^-). (b) Overlap of miltefosine effect on cell viability and on ROS accumulation in *L. infantum* after 24 h of drug pressure. Data shown represents mean ± SEM from three independent biological replicates.

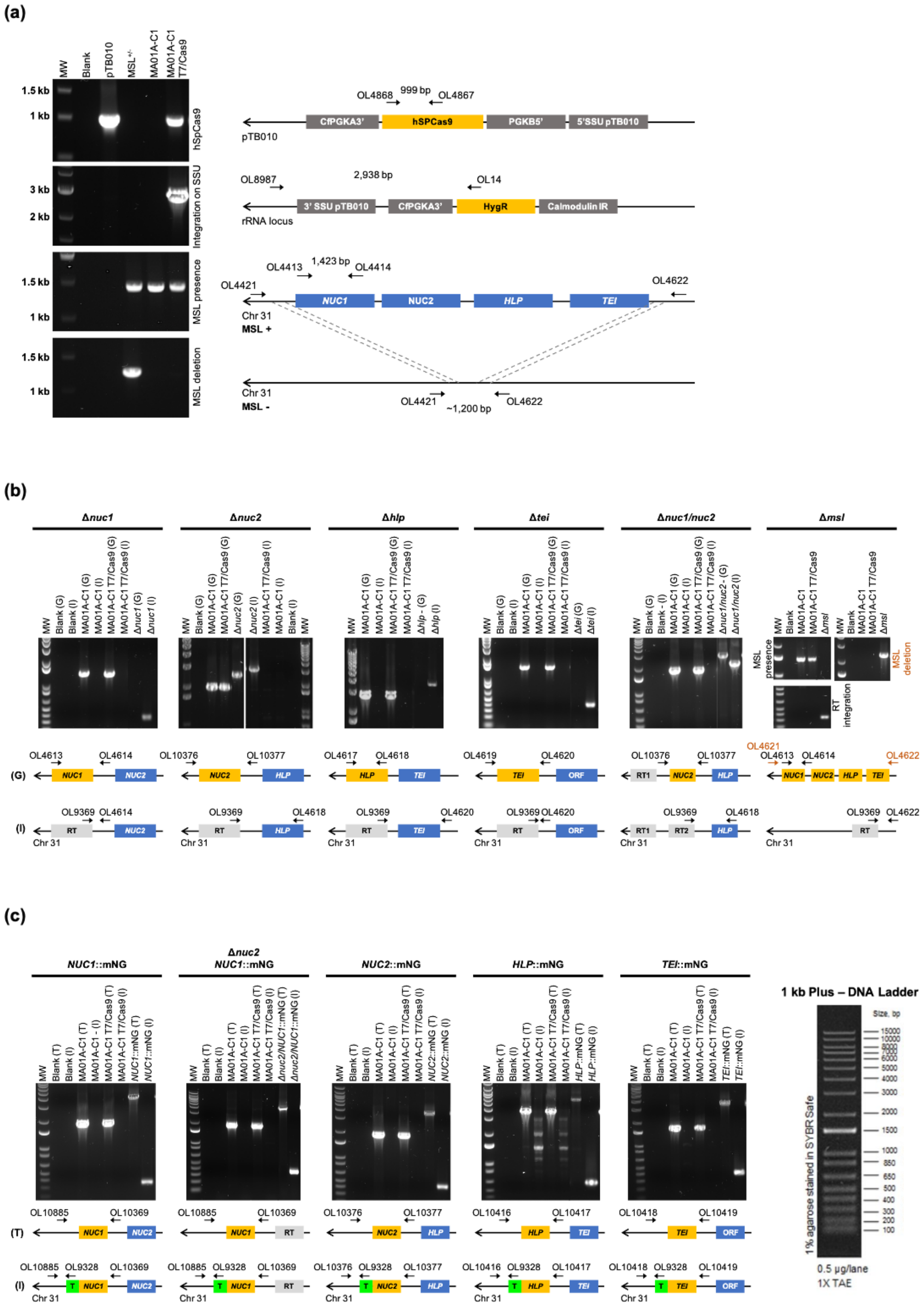

Supplementary Figure 3: PCR screening of the cell lines generated.

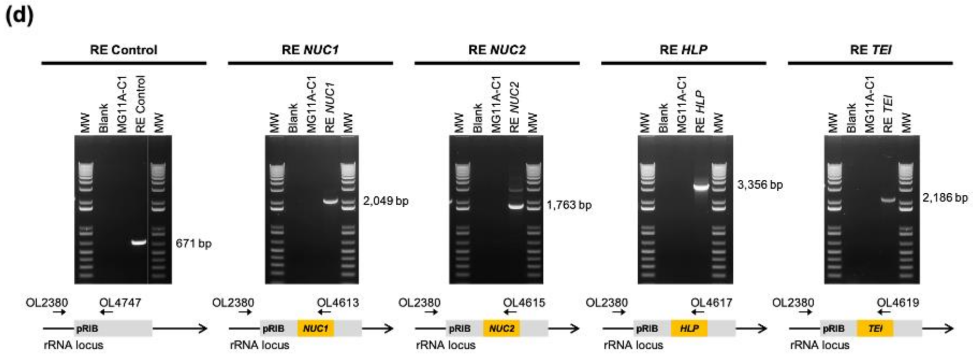

Supplementary Figure 3: PCR screening of the cell lines generated. Cell lines generated in this study were all screened by PCR and each PCR strategy used, including the set of oligonucleotides (OL), is shown. (a) Generation of *L. infantum* expressing T7 RNA polymerase and Cas9 endonuclease. (b) knock out (KO) cell lines generated by CRISPR-Cas9. The target gene is highlighted in yellow, whereas the repair template (RT) and neighbouring ORFs are indicated by grey and blue boxes, respectively. The results of the PCRs to screening for the presence of the target gene (G) and for the integration (I) of RT are interpreted as follow: (i) Δ*nuc1*, the presence of the target gene is indicated by a PCR product of 1423 bp whereas its absence by a negative PCR. Integration of RT is shown by a PCR product of 408 bp; (ii) Δ*nuc2*, the presence of the target gene is indicated by a PCR product of 1938 bp whereas its replacement by RT carrying puromycin (PAC) or blasticidin (BSD) resistance genes by a PCR product of 2436 bp and 2305 bp, respectively. Integration of RT is shown by a PCR product of 3177 bp; (iii) Δ*hlp*, the presence of the target gene is indicated by a PCR product of 2727 bp whereas its absence by a negative PCR. Integration of RT is shown by a PCR product of 4331 bp; (iv) Δ*tei*, the presence of the target gene is indicated by a PCR product of 1562 bp whereas its absence by a negative PCR. Integration of RT is shown by a PCR product of 423 bp; (v) Δ*nuc1*/*nuc2*, the Δ*nuc1* cell line, carrying only PAC resistant mark (RT1), was used to generate Δ*nuc1*/*nuc2*. The screening to check deletion of NUC2 was performed as the one mentioned for Δ*nuc2*; (vi) Δ*msl*, the presence of MSL locus was checked by a PCR product of 1423 bp, corresponding to the presence of NUC1, whereas its absence by a negative PCR. The deletion of MSL was also checked by a PCR flanking the locus, which generated a fragment of 2412 bp or 2289 bp if the locus was replaced by RT carrying PAC or BSD genes, respectively. Integration of RT is shown by a PCR product of 340 bp. (c) C-terminal tagged cell lines generated by CRISPR-Cas9. The target gene is indicated in yellow and the mNeonGreen tag (T) in green. The PCRs used to show that all alleles were tagged were designed using a set of primers flanking the target gene and are interpreted as follow: (i) NUC1::mNG, tagged and untagged alleles show a PCR product of 4348 bp and 1929 bp, respectively. Integration in the right position is shown by a PCR product of 537 bp. (ii) NUC2::mNG, tagged and untagged alleles show a PCR product of 4,390 bp and 1,938 bp, respectively. Integration in the right position is shown by a PCR product of 576 bp. (iii) HLP::mNG, tagged and untagged alleles show a PCR product of 6194 bp and 3699 bp, respectively. Integration in the right position is shown by a PCR product of 780 bp. (iv) TEI::mNG, tagged and untagged alleles show a PCR product of 4206 bp and 1778 bp, respectively. Integration in the right position is shown by a PCR product of 601 bp. (d) Cell lines re-expressing (RE) MSL genes individually on rRNA locus of the *L. infantum* isolate (MG11A-C1), that lacks this locus on chromosome 31. The re-expression was performed using the pRIB vector. SSU, rRNA locus on *Leishmania* spp.; MSL, miltefosine susceptible locus; NUC1, LINF_310031200; NUC2, LINF_310031300; HLP, LINF_310031400; TEI, LINF_310031500.

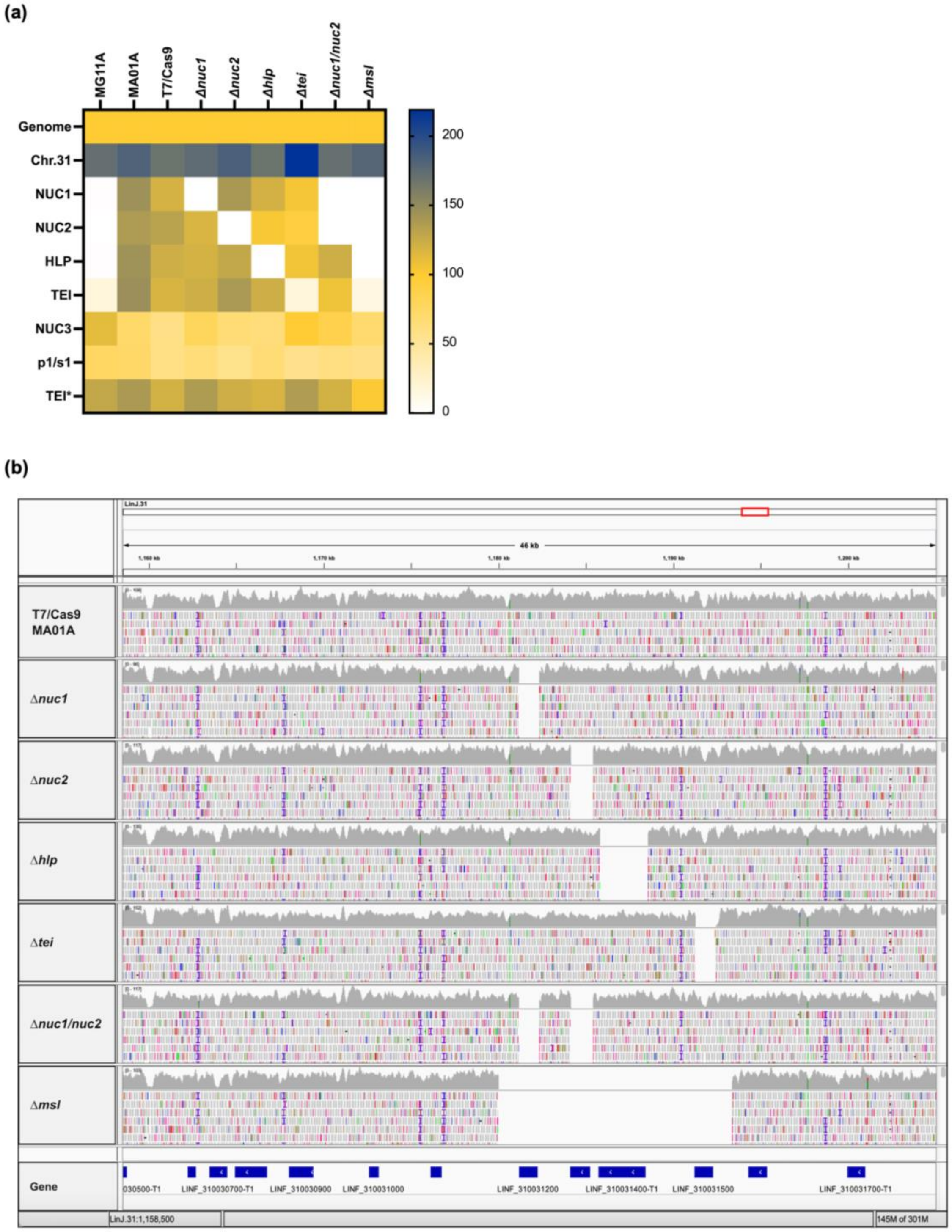

Supplementary Figure 4: Whole Genome Sequence analysis of *L. infantum* isolates/mutants. (a) Aneuploidy in natural populations and mutants. The heatmap shows the copy-number status of chromosome 31, the MSL genes (*NUC1*, LINF_310031200; *NUC2*, LINF_310031300; *HLP*, LINF_310031400; and *TEI*, LINF_310031500) and the MSL paralog (*NUC3*, LINF_120009100; p1/s1, LINF_300020200) and orthologue (*TEI**, LINF_310030700) genes. (b) Integrative genomics viewer (IGV, v.2.7.2) of the Illumina whole genome sequencing reads from △*nuc1*, △*nuc2*, △*hpl*, △*tei*, △*nuc1/nuc2*, and △*msl* mutants aligned against the *L. infantum* reference genome JPCM5. Visual inspection of the aligned reads in a genome browser of the sequencing coverage of chromosome 31 region that contain the genes from the MSL locus. Generally, a lack of coverage in sequenced isolates must be interpreted carefully, as it can often represent a region that does not replicate or map well. Visual inspection of the aligned reads in a genome browser, however, supported the calculated predictions with the presence of reads that are split over the predicted deletion indicating that this is a genuine deletion and not a poorly sequenced region. The WGS data generated for all mutants were added to the previous SRA project ID PRJNA494801.

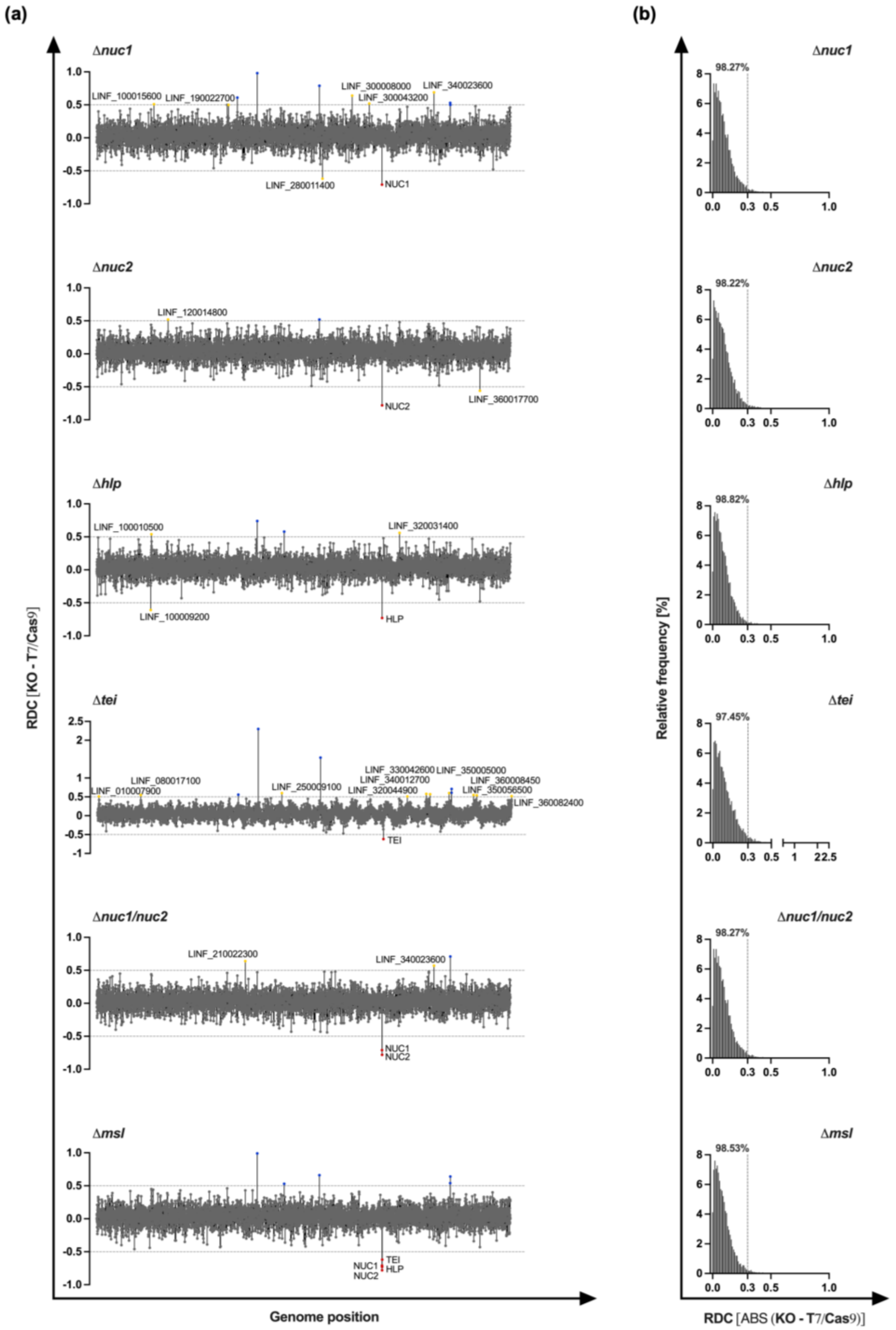

Supplementary Figure 5: Read depth coverage differences between MSL mutants and *L. infantum* T7/Cas9. (a) Read depth coverage (RDC) of each coding sequence (CDS) were estimated, normalized per chromosome RDC, and compared between the MSL knockout mutants and T7/Cas9. All CDS with difference [mutants - T7/Cas9] higher than 0.5 RDC, which corresponds to approximately one copy per haploid genome, are highlighted in yellow or blue (CDS that RDC varied in more than one MSL knockout line). MSL genes are highlighted in red. Each line of the X axis corresponds to a CDS, ordered by its genome position from the smallest to the largest chromosome. (b) Histogram plot of the absolute RDC differences between MSL knockout lines and T7/Cas9. The accumulated frequency for absolute RDC difference until 0.3 is indicated in each plot [%].

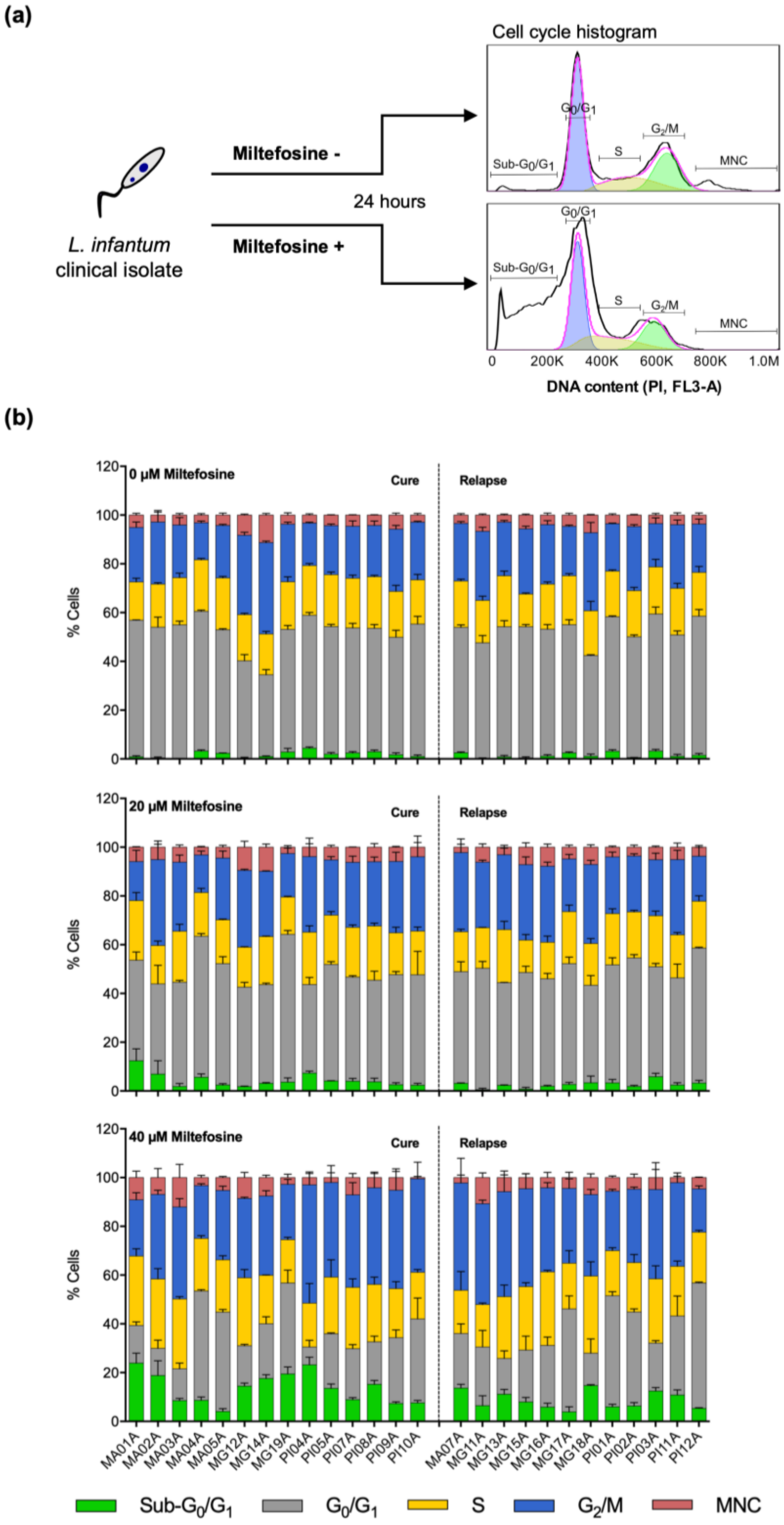

Supplementary Figure 6: Effect of miltefosine on the cell cycle of *L. infantum* promastigotes. (a) experimental outline of the cell cycle analysis. Cell cycle analysis of the 26 *L. infantum* isolates (14 from group cured and 12 from group relapsed) was carried out using propidium iodide staining after 24 h of miltefosine pressure. The FlowJo v.10.6.2 cell cycle algorithm (Dean-Jett-Fox model) was used to measure the percentage of cells in each cell cycle stage. (b) Percentage of cell in each cell cycle stage. Data shown represents mean ± SEM from three independent biological replicates. G, gap phase; S, synthesis phase; M, mitosis phase; MNC, multi nuclear cells; Sub-G_0/G_1 phase indicate cells with degraded DNA, cells containing less DNA than typically observed in G_0/G_1.

(a)

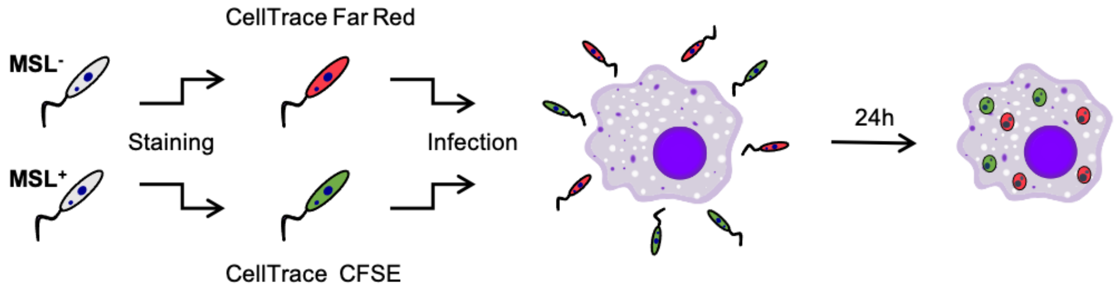

(b)

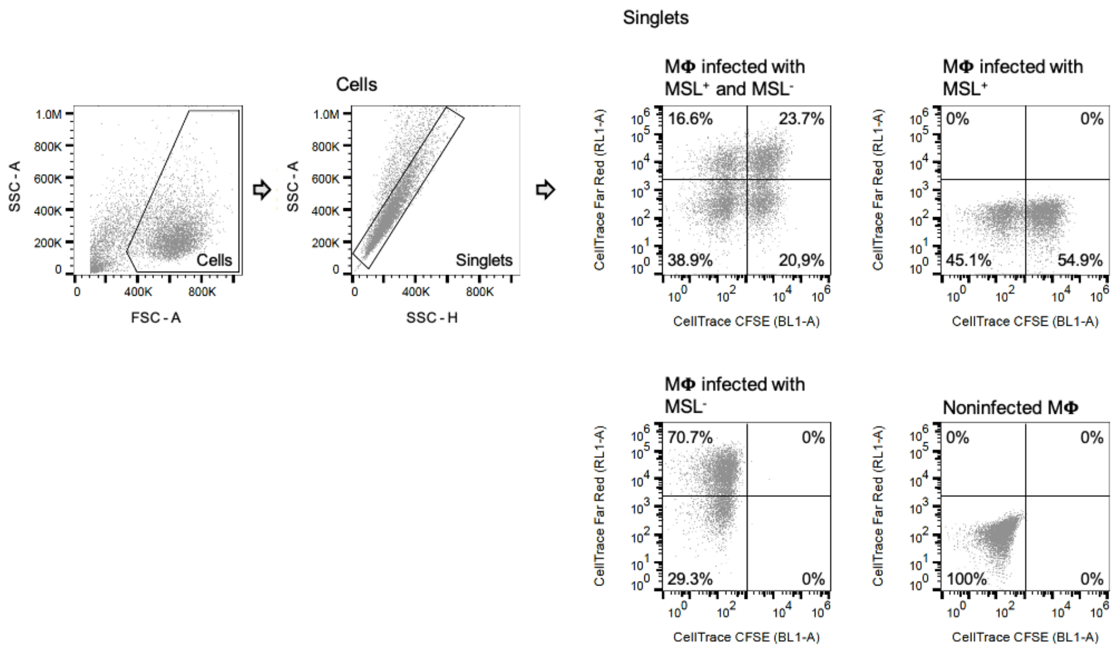

Supplementary Figure 7: Multiple *Leishmania* infection in RAW264.7 macrophage. (a) experimental draw to show if RAW264.7 macrophage can be simultaneously infected with more than one parasite genotype. *L. infantum* MSL^+ and MSL^- were individually stained with CellTrace CFSE and Far red, respectively, mixed and then used to infect RAW264.7. After 24 h of infection the macrophages were analysed by flow cytometry. (b) Flow cytometry plots and gate strategy used to analyse the intracellular infection of RAW264.7 macrophage by *Leishmania* parasites: MSL^+ and MSL^- simultaneously; MSL^+; MSL^-; and a noninfected control.

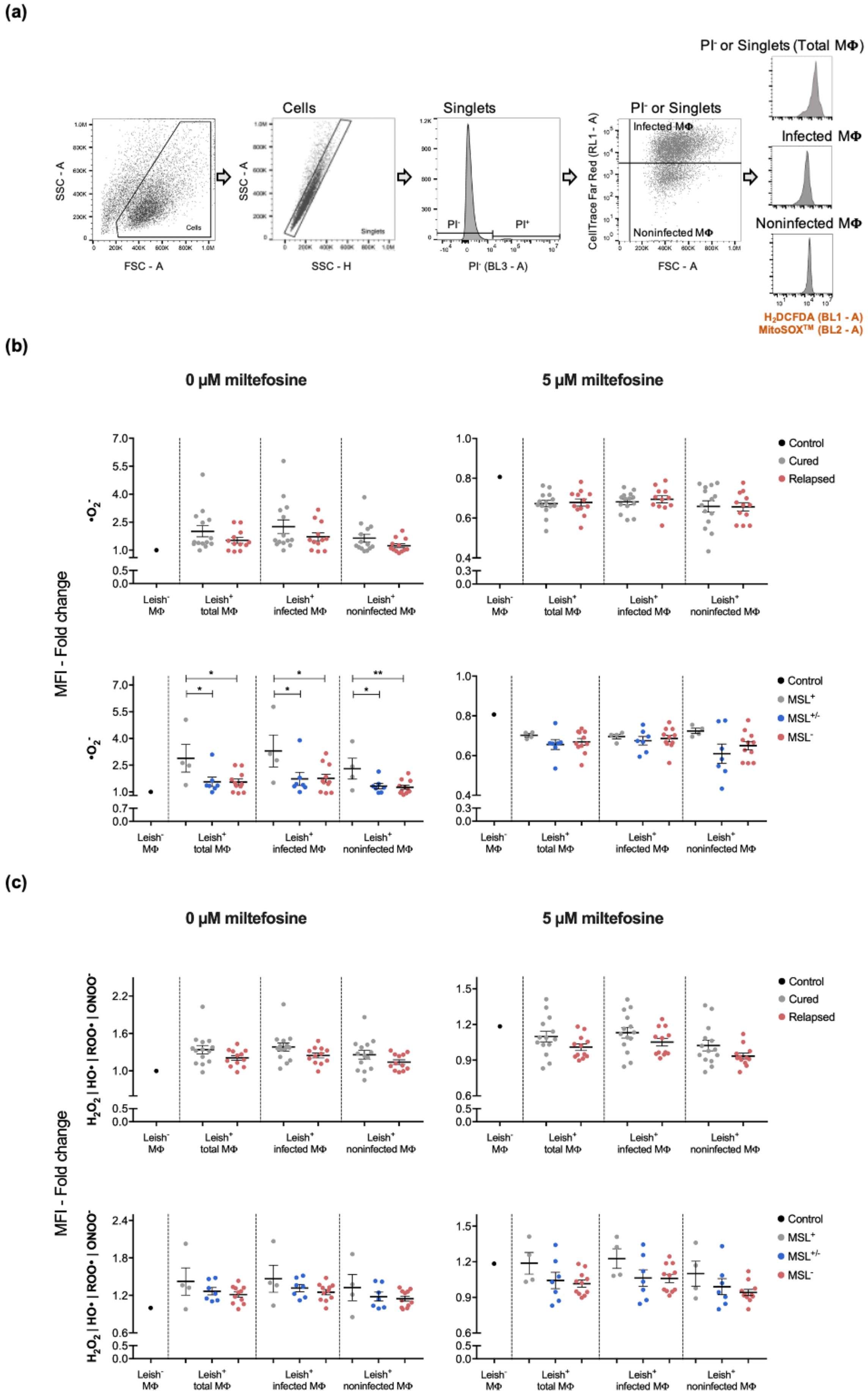
(a)
PI- or Singlets (Total MΦ)
Cells
Singlets
PI- or Singlets
Infected MΦ
Noninfected MΦ
SSC - A
FSC - A
SSC - H
PI- (BL3 - A)
CellTrace Far Red (RL1 - A)
H2DCFDA (BL1 - A)
MitoSOX™ (BL2 - A)
(b)
0 µM miltefosine
5 µM miltefosine
MFI - Fold change
•O2-
Control
Cured
Relapsed
MSL+
MSL+/-
MSL-
Leish- MΦ
Leish+ total MΦ
Leish+ infected MΦ
Leish+ noninfected MΦ
(c)
0 µM miltefosine
5 µM miltefosine
H2O2 | HO• | ROO• | ONOO-

Supplementary Figure 8: Intracellular level of ROS into RAW264.7 cells challenged with different *L. infantum* isolates. (a) RAW264.7 cells challenged for 24 h with *L. infantum* (26 isolates: 14 from group cured and 12 from group relapsed. The isolates were also sorted by their MSL genotype/clinical outcome: 4 MSL^{+}/cured, 7 $MSL^{+/-}$/cured, and 11 MSL^{-}/relapsed) were subsequently treated with 0 or 5 µM of miltefosine for an additional 24 h. (a) Gate strategy used to analyse the intracellular level of ROS into different populations. The level of H_2O_2, HO•, ROO• and $ONOO^-$ and mitochondrial superoxide ($•O_2^-$) for total macrophage (total MΦ) were measured on PI^- and singlets populations, respectively. (b) The reactive oxygen species were measured using the probes H_2DCFDA (H_2O_2, HO•, ROO• and $ONOO^-$) and MitoSOX^{TM} ($•O_2^-$). In the absence of miltefosine, the cells were normalized by the untreated not challenged RAW264.7 cells (Control $Leish^-$ MΦ), whereas the miltefosine treated RAW264.7 cells (challenged or not with *L. infantum* parasite) were normalized by their untreated pair. Each dot represents the mean of individual *L. infantum* isolate from three independent experiments. Mean and ± SEM for the groups is shown in black. P values were calculated using unpaired two-tailed Student's t-tests comparing the group cured with relapsed; and using One-way ANOVA test comparing the groups MSL^{+}, $MSL^{+/-}$ and MSL^{-} (* p-value <0.05; ** p-value < 0.01). MΦ, macrophage; $Leish^-$, not challenged with *L. infantum* parasite; $Leish^+$, challenged with *L. infantum* parasite.

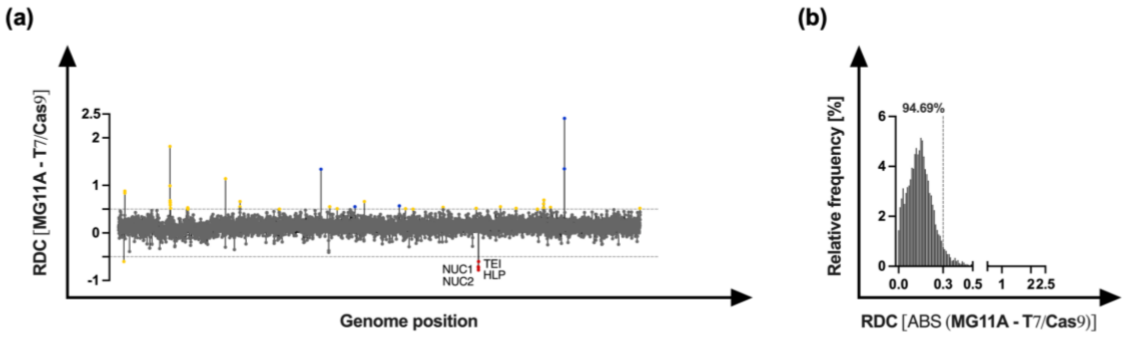

Supplementary Figure 9: Read depth coverage differences between *L. infantum* MG11A and T7/Cas9. (a) Read depth coverage (RDC) of each coding sequence (CDS) were estimated, normalized per chromosome RDC, and compared between MG11A and T7/Cas9. All CDS with difference [MG11A - T7/Cas9] higher than 0.5 RDC, which corresponds to approximately one copy per haploid genome, are highlighted in yellow or blue (CDS that RDC also varied in MSL knockout lines). MSL genes are highlighted in red. Each line of the X axis corresponds to a CDS, ordered by its genome position from the smallest to the largest chromosome. (b) Histogram plot of the absolute RDC differences between MG11A and T7/Cas9. The accumulated frequency for absolute RDC difference until 0.3 is indicated in each plot [%].

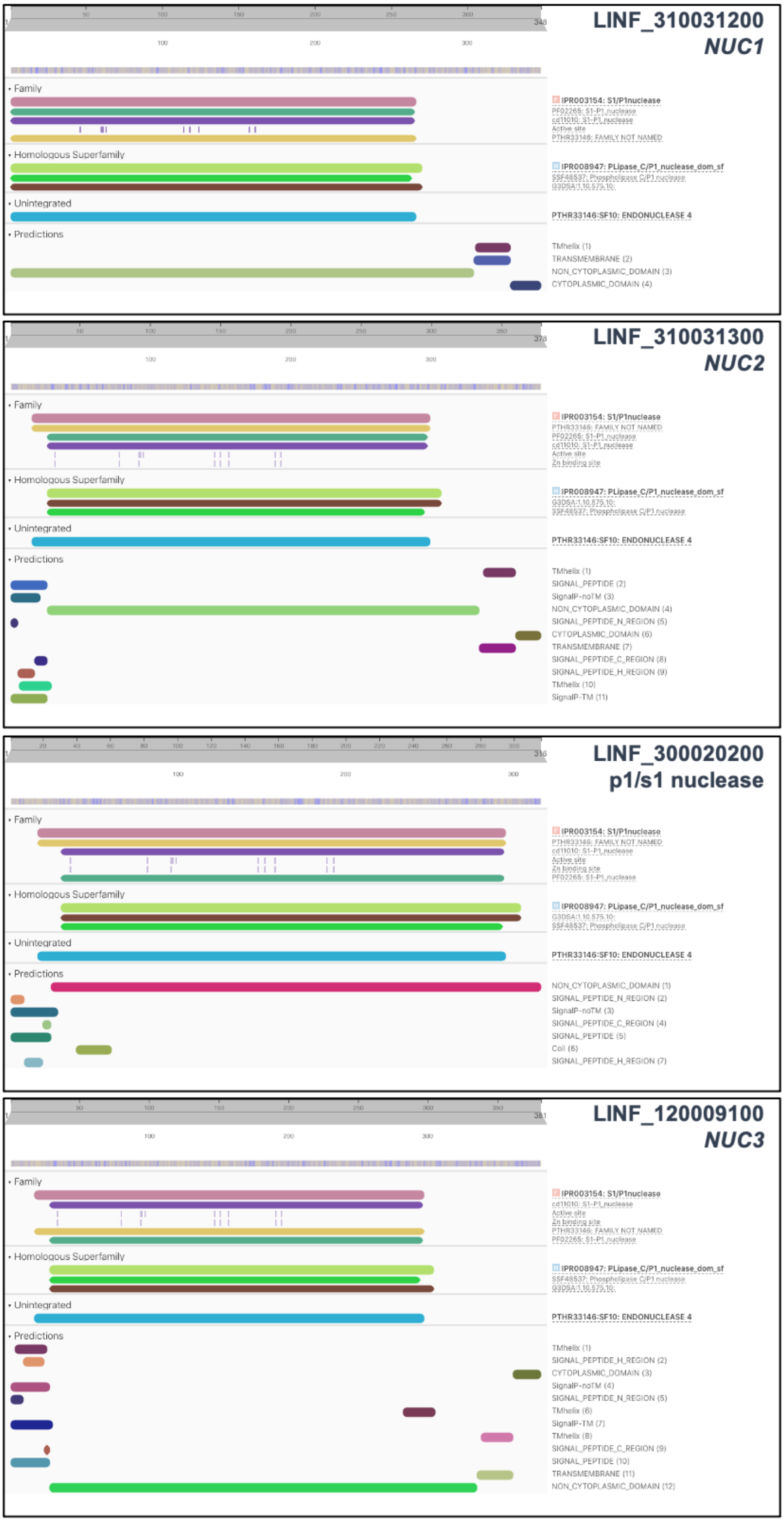

Supplementary Figure 10: In-silico protein domain analysis for *L. infantum* 3'nucleotidase/nuclease. Protein domain prediction was performed on online InterProScan tool.

(a)

```
CLUSTAL O(1.2.4) multiple sequence alignment


LINF_300020200      MPA--LVGLRLPLTVLCLLVLSSALCVTEALGWGCVGHMLLAEIARRQLDDKNKEKIDAM      58
LINF_120009100      MAAFAFSAHALLAAVIAML----LLLALPTQAWWDKGHMCIAEIARRNLKPNVQAKVQAC      56
LINF_310031200      --------------------------------------MAVALIAQRHMDPKLVKKANAA      22
LINF_310031300      MARARF--LQLLLVTLTLL----STAALPVSAWWSKGHMSVALIAKRHMGASLVEKAELA      54
                                                          * :* **:*::  .   * :

LINF_300020200      AEVFAQSGPFPSSPDMVQAACWADDVKLWRQRAMGSWHYFDAPYNPENIN-I-TDAIATV      116
LINF_120009100      ADVLNKIGPFPKSTNIVELGPWADDLKSMGLYTMSTWHFIDTIYNP-QDVKVTINPVEIV      115
LINF_310031200      AKVFSLAGPFPKSPDMVQLGPWADDLLESGLKTNFNWHFITAPYYPDADFTLELSPAQTV      82
LINF_310031300      AKVLSFSGPYPKSPDMVQTAPWADDIKTIGLKTLSTWHYITTPYYTDEDFTLDVSPVQTV      114
                    *.*:   **:*.* ::*: . ****:      :  .**:: : *       :  .    *

LINF_300020200      NAVTVSRNMISALKNTKAPLYMLNFAWANLVHIFGDLHQPLHTISRYSSEYPHGDKGGNL      176
LINF_120009100      NVASVIPMLISAITSPAATSDIITTSVANLIHFVGDIHMPLHSADLFSPEYPLGDLGGNK      175
LINF_310031200      NAASVIPMLESAIRKTTATTEIITQCLAFMVHLFGDIHQPLHNANLLSDEYPLGDYGGNA      142
LINF_310031300      NVASVIPMLQTAIEKPTANSDVIVQSLALLLHFMGDIHQPLHNVNLFSNQYPESDLGGNK      174
                    *..:*   : :*: .  *   ::  . * ::*:.**:* ***. .  * :** .* ***

LINF_300020200      IQVMV---GRKSLRLHALWDNICTGTPPR-YQRPLSYTDLFALAATADRLLETYIF--PE      230
LINF_120009100      QIVIVNESAGTSMKLHAFWDSMCEGPQNN-AVRPLDKDAYAELSAFVDNLVKSYSF--TE      232
LINF_310031200      QMVTI-DSNGTKMLLHAYWDSMAEGPASVGYPRPLSKDAYEDLKAFVDYLEATYAGNLTT      201
LINF_310031300      QLVVI-DSKGTKMLLHAYWDSMAEGKSGEDVPRPLSEADYDDLNNFADYLEATYASTLTD      233
                      * :     ..: *** **.:. *       ***.      *   .* *  :*

LINF_300020200      ALRTLVDVMAIHEESHMFAVNTSYPGVTPGATLSEAYLARCKRVAEARLTLGGYRLGYLL      290
LINF_120009100      EQMMMTNSTIMAAESYELAVKNVYPGISDRTVLSETYKANGKILAAGRVTLAGYRLATIL      292
LINF_310031200      AEKNLQNTTAIRNEGYELAIKYAFPGASNGATLSNEYKTNAKKISERQVLLAGYRLAKML      261
LINF_310031300      KEKNLVDTTEISKETFDLALKYAYPGADNGATLSDEYKTNAKKISERQVLLAGYRLAKML      293
                        : :   :  * . :*::  :**    :.**: * :. * ::  :: *.****. :*

LINF_300020200      NTLLSSIHVDEATLEAYR--AARPK--RGA------------------------------      316
LINF_120009100      NTALAGVSLDTIMNGTKHMQDEVEVTHTGDTYNYYAFSGVESGAAAGIFLSSFAIGCLLA      352
LINF_310031200      NTTLKSVSMDTILQGLGNIQAEVPRLDNTTIGHTFQEKGLSTGLTVGIAIALFIAGVLIS      321
LINF_310031300      NTTLKSVSMDTILQGLKSIQSEVDTENKAEVHNHYDQKGISAAVTAIVAVALFIAGIIIA      353
                    ** * .: :*

LINF_300020200      -----------------------------      316
LINF_120009100      TAVVLAALYMRRGSSKDERAAAASASHGI      381
LINF_310031200      TVVVLFLNRPRLVRSDYQRCEPIALD--L      348
LINF_310031300      TLVVLALKCYLPKRDRFGSYEHVAL----      378
```

(b)

% Identity Matrix	LINF_300020200	LINF_120009100	LINF_310031200 (NUC1)	LINF_310031300 (NUC2)
**LINF_300020200**	100.00	36.01	38.57	35.16
**LINF_120009100**	36.01	100.00	45.64	43.32
**LINF_310031200**	38.57	45.64	100.00	63.01
**LINF_310031300**	35.16	43.32	63.01	100.00

Supplementary Figure 11: ***Leishmania infantum*** **3'Nucleotidase/Nuclease protein sequences Analysis.** (a) Multiple sequence alignment performed with Clustal Omega (1.2.4). (b) Percent Identity Matrix - created by Clustal2.1. NUC1 (LINF_310031200); NUC2 (LINF_310031300); P1/S1 (LINF_300020200); NUC3 (LINF_120009100).
