# Supplementary material for: 3′Nucleotidase/nuclease is required for Leishmania infantum clinical isolate susceptibility to miltefosine
Source: eBioMedicine. 2022 Nov 30;86:104378. doi: 10.1016/j.ebiom.2022.104378 (PMC9713291; doi:10.1016/j.ebiom.2022.104378)
Supplement: Supplementary File S1 [file mmc2.docx]

**Caption for supplementary material**

**Supplementary Table 1:** Sequence of oligonucleotides.

**Supplementary Table 2:** Read depth coverage differences between MSL mutants/MG11A and *L. infantum* T7/Cas9.

**Supplementary Table 3:** Summary of variants identified in coding sequence region of the *L. infantum* knockout mutants against T7/Cas9.

**Supplementary Table 4:** Summary of variants identified in coding sequence region of the *L. infantum* MG11A against T7/Cas9.

**Supplementary Figure 1:** Map of the plasmid pTB010.

**Supplementary Figure 2:** Concentration and time effect of miltefosine in ROS accumulation and cell viability on promastigote stage of *L. infantum* parasite.

**Supplementary Figure 3:** PCR screening of the cell lines generated.

**Supplementary Figure 4:** Whole Genome Sequence analysis of *L. infantum* isolates/mutants.

**Supplementary Figure 5:** Read depth coverage differences between MSL mutants and *L. infantum* T7/Cas9.

**Supplementary Figure 6:** Effect of miltefosine on the cell cycle of *L. infantum* promastigotes.

**Supplementary Figure 7:** Multiple *Leishmania* infection in RAW264.7 macrophage.

**Supplementary Figure 8:** Intracellular level of ROS into RAW264.7 cells challenged with different *L. infantum* isolates.

**Supplementary Figure 9:** Read depth coverage differences between *L. infantum* MG11A and T7/Cas9.

**Supplementary Figure 10:** In-silico protein domain analysis for *L. infantum* 3’nucleotidase/nuclease.

**Supplementary Figure 11:** *Leishmania infantum* 3’Nucleotidase/Nuclease protein sequences Analysis.
